# Nanotechnological applications of marine algae derived neurotoxins spanning harmful algal blooms and therapeutic innovations

**DOI:** 10.1186/s11671-026-04767-4

**Published:** 2026-06-28

**Authors:** Morteza Golbashirzadeh

**Affiliations:** Marine Medicinal Plants Research Center, Chabahar University of Medical Science, Chabahar, Iran

**Keywords:** Neurotoxin biosynthesis, Harmful algal blooms (HABs), Marine algae-derived neurotoxins, Nanotechnology for algae toxins, Neurotoxin effects

## Abstract

Marine algae-derived neurotoxins, commonly associated with harmful algal blooms (HABs), represent a paradox in marine and biomedical sciences. While traditionally studied for their ecological disruptions and health hazards, these bioactive compounds possess unique biochemical properties with promising therapeutic and technological applications. By summarizing current knowledge and identifying gaps in research, this review systematically examines the biological significance of marine algae, elucidates the mechanisms underlying neurotoxin production, and assesses their biomedical relevance. Furthermore, it explores nanotechnology as a transformative approach for harnessing neurotoxins in drug delivery, biosensing, and theranostics. Particular emphasis is placed on nano-biosensors for precise neurotoxin detection, nanomaterial-based mitigation strategies for HABs, and the use of neurotoxin-loaded nanocarriers in targeted pharmacological interventions. Ethical and environmental considerations surrounding neurotoxin utilization are critically analyzed to ensure sustainable applications. By synthesizing current knowledge and identifying gaps in research, this review hypothesizes that marine algae-derived neurotoxins, when incorporated into nanosystems, can serve as multifunctional agents in precision medicine, neuropharmacology, and diagnostic imaging. Through an interdisciplinary perspective bridging marine biology, toxicology, and nanoscience, this work aims to provide a structured framework for future research, fostering innovation at the intersection of marine biotechnology and nanomedicine.

## Introduction

Marine algae serve as foundational components of aquatic ecosystems, playing a pivotal role in primary productivity, nutrient cycling, and oxygen generation [1]. These photosynthetic organisms contribute significantly to global carbon sequestration while supporting complex marine food webs. Their diverse biochemical profiles allow them to adapt to varied environmental conditions, ranging from open oceanic waters to nutrient-rich coastal systems [2, 3].

Despite their ecological importance, certain algae species can undergo excessive proliferation, leading to harmful algal blooms (HABs). These events, often triggered by rising ocean temperatures, nutrient influx, and hydrodynamic shifts, can have devastating consequences for marine biodiversity, fisheries, and human health [4]. Because harmful algal blooms (HABs) occur unpredictably, ongoing ecological surveillance is essential. These events disrupt aquatic food chains and generate potent neurotoxins that accumulate in marine organisms, creating significant risks for both human consumption and long‑term environmental sustainability.

However, the problem lies in the dual nature of marine algae-derived neurotoxins: while they are hazardous compounds responsible for ecological damage and human intoxications, they also possess unique biochemical properties that can be harnessed for biomedical innovation. Therefore, although HABs are widely recognized for their detrimental effects, marine algae also harbor untapped potential in biotechnology [5]. Their secondary metabolites, including neurotoxins, offer opportunities for applications beyond environmental hazards, particularly in drug development, nanomedicine, and biosensing technologies. As scientific interest in algae-derived biomolecules grows, there is an urgent need to explore both their adverse effects and their transformative potential in biomedical innovation [5]. This gap thus presents an opportunity to reframe neurotoxins not only as threats but also as valuable biomolecules with translational potential, making the topic both scientifically significant and attractive to a broad readership.

Neurotoxic compounds produced by marine algae, including saxitoxins, brevetoxins, and domoic acid, exhibit highly specific biochemical interactions that affect neurological function [6]. These toxins primarily act on voltage-gated ion channels and neurotransmitter receptors, leading to severe disruptions in neuronal communication. While their toxicity poses significant concerns for marine ecosystems and human health, recent advances in neuroscience have recognized their potential utility as research tools and therapeutic agents. For instance, saxitoxin functions as a selective sodium channel blocker, making it a valuable tool for studying neuronal excitability and nerve conduction disorders. Likewise, brevetoxins modulate sodium channel kinetics, offering insight into neuroinflammatory mechanisms and disease pathology [7]. Domoic acid, known for its glutamate receptor agonist activity, has been utilized to model excitotoxicity in Alzheimer’s disease and epilepsy research. Consequently, The ability of these neurotoxins to precisely target neural pathways has opened new avenues in pharmacology, particularly in drug development for neurodegenerative conditions, chronic pain management, and synaptic modulation therapies [8].

Despite their therapeutic promise, challenges remain in regulating neurotoxin bioactivity and ensuring controlled application in medical contexts. Continued research must therefore focus on refining delivery mechanisms and minimizing unintended toxicity while optimizing pharmacological benefits.

Nanotechnology has emerged as a transformative tool for enhancing the functionality and safety of marine neurotoxins in biomedical applications [9]. By incorporating neurotoxin molecules into nanoscale carriers, researchers can regulate their bioavailability, improve their therapeutic precision, and mitigate toxicity concerns. Nanocarriers such as liposomes, polymeric nanoparticles, and metallic nanostructures have demonstrated potential for encapsulating and delivering neurotoxins in controlled doses, ensuring optimal pharmacological outcomes while preventing off-target effects [10, 11].

Beyond drug delivery, nanotechnology plays a crucial role in biosensing applications, allowing for the rapid and sensitive detection of marine neurotoxins in seafood, water samples, and environmental monitoring systems. Nano-enabled biosensors utilize highly specific recognition elements to bind neurotoxic molecules, generating quantifiable signals that facilitate timely toxin detection. These platforms are instrumental in public health initiatives, mitigating seafood contamination risks while improving HAB surveillance strategies [12, 13].

In theranostics the integration of therapeutic and diagnostic functions—marine neurotoxins encapsulated within nanocarriers have demonstrated potential for precision medicine. The significance of this review lies in addressing the current gap: despite extensive research on HABs and neurotoxin biosynthesis, systematic exploration of their integration within nanotechnological frameworks is still in its infancy. This work aims to provide a structured perspective on how marine algae‑derived neurotoxins can be transformed from ecological hazards into multifunctional agents for biomedical innovation. By critically analyzing existing knowledge, identifying research gaps, and proposing interdisciplinary strategies, this review seeks to attract attention to an emerging frontier at the intersection of marine biology, toxicology, and nanomedicine.

## Harmful algal blooms (HABs) and neurotoxin production

### Overview of HABs and their global significance

Harmful algal blooms (HABs) are rapid proliferations of algae, particularly microalgae, in aquatic ecosystems that lead to significant environmental and health impacts. While algal blooms occur naturally, human activities such as nutrient pollution, climate change, and water temperature fluctuations have intensified their frequency and severity [14].

From a technical perspective, HABs are characterized by high cell densities, often exceeding 10^6^ cells per liter, which can result in hypoxic or anoxic conditions in affected waters. The proliferation is typically driven by eutrophication, where excess nitrogen and phosphorus inputs accelerate algal growth. Physical parameters such as water column stratification, salinity gradients, and light penetration also play critical roles in bloom initiation and persistence. Consequently Monitoring programs often rely on chlorophyll-a concentration as a proxy for algal biomass, with thresholds above 10 µg/L commonly used to indicate bloom conditions [15].

Globally, HABs pose a major ecological and economic concern. In coastal waters, they disrupt marine food webs, deplete oxygen levels, and contribute to mass fish kills. Moreover, they affect industries reliant on seafood, tourism, and recreation [16]. Economic losses from HABs are estimated in the billions annually, due to fisheries closures, aquaculture damage, and healthcare costs associated with toxin exposure [17]. Some species involved in HABs produce potent neurotoxins that accumulate in seafood and pose direct threats to human health. These toxins, such as saxitoxins and domoic acid, are responsible for severe neurological disorders when consumed, leading to widespread health warnings and regulatory interventions [18]. In addition to direct toxicity, In addition to direct toxicity, HABs can cause secondary impacts such as biofouling of aquaculture systems, alteration of benthic habitats, and long-term shifts in ecosystem structure. Finally Technical advances in remote sensing, molecular diagnostics, and in situ biosensors are increasingly being applied to detect HABs early, quantify toxin concentrations, and predict bloom trajectories. Such tools are critical for mitigating risks and informing management strategies at regional and global scales [19].

### Biochemical pathways of neurotoxin synthesis in marine algae

The biosynthesis of neurotoxins in marine algae is a highly intricate and evolutionarily refined process, governed by specialized metabolic pathways that vary significantly among species. For example, Toxin-producing microalgae, including *Alexandrium*, *Karenia*, and *Pseudonitzschia*, employ diverse enzymatic mechanisms to convert primary metabolites into potent neurotoxic compounds. These pathways are not only species-specific but are also influenced by a complex interplay of genetic, biochemical, and environmental factors [20, 21].

**Fig. 1:**
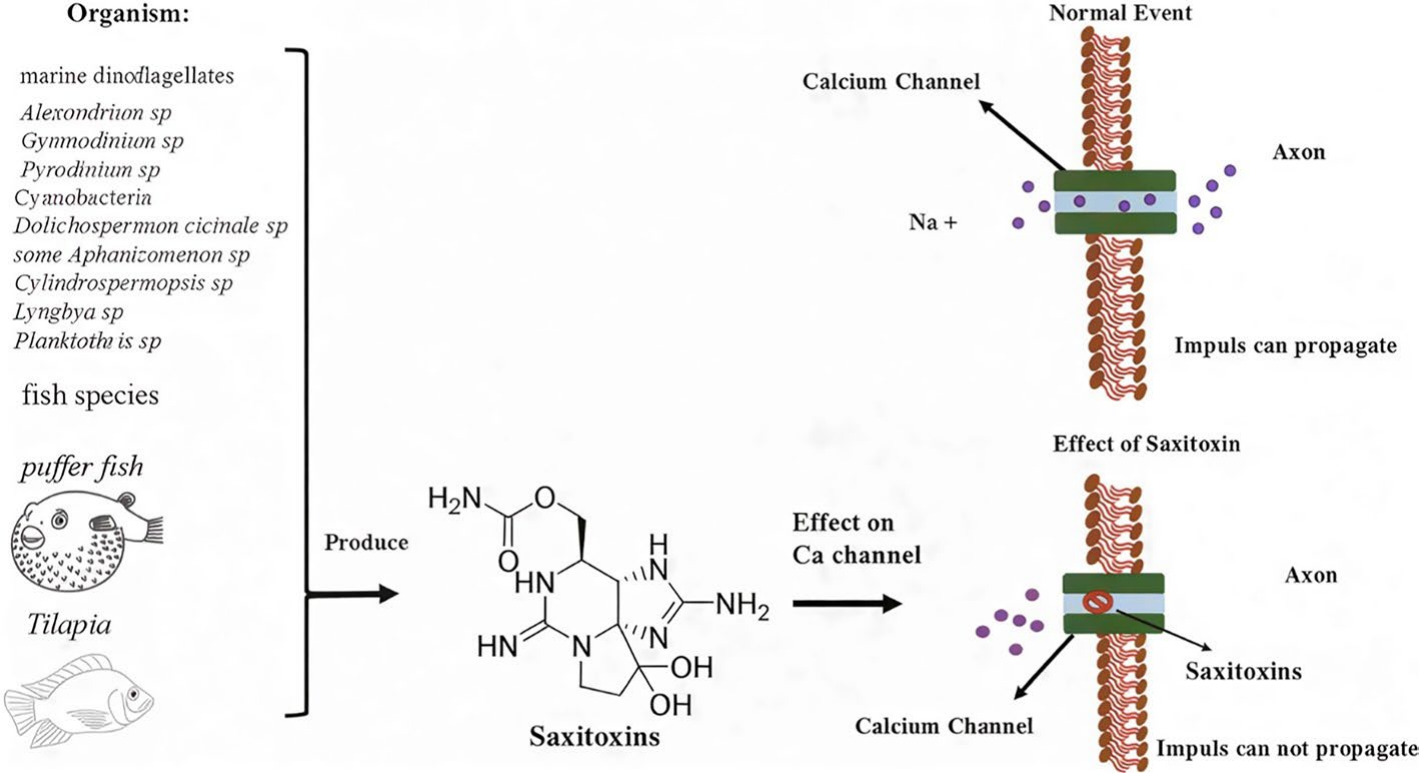
Representative overview of saxitoxin-producing organisms and the neurotoxic effects of saxitoxin on calcium channels. The figure illustrates key cyanobacterial genera (e.g.,*Aphanizomenon, Dolichospermum, Cylindrospermopsis*) and marine dinoflagellates (e.g., *Alexandrium, Gymnodinium*) known to biosynthesize saxitoxin. Saxitoxin, a potent neurotoxin, is shown interacting with voltage-gated calcium channels, contributing to disrupted ion flux and impaired neuronal signaling. This mechanism complements its well-documented blockade of sodium channels, highlighting its broader impact on excitable cells

A well-studied example is the synthesis of saxitoxins paralytic shellfish toxins predominantly produced by dinoflagellates of the genus *Alexandrium* [22]. The biosynthetic pathway begins with arginine and other amino acid precursors, which undergo a series of enzymatic transformations, including methylation, oxidation, and cyclization, ultimately yielding a suite of structurally related neurotoxic alkaloids. Figure 1 provides an overview of saxitoxin-producing organisms, including *cyanobacterial genera* such as *Aphanizomenon, Dolichospermum*, and *Cylindrospermopsis*, as well as marine dinoflagellates like *Alexandrium* and *Gymnodinium*. The figure further highlights the neurotoxic mechanism of saxitoxin, depicting its interaction with voltage-gated calcium channels, where it contributes to disrupted calcium ion flux and altered neuronal communication. This complements its established role in blocking voltage-gated sodium channels, emphasizing its broader influence on excitable cell signaling.

Similarly, a neurotoxin associated with amnesic shellfish poisoning synthesized by diatoms of the genus *Pseudo- nitzschia* via polyketide biosynthesis. This process involves the stepwise assembly of acetate and malonate units, followed by subsequent modifications that confer the molecule’s neuroactive properties (Fig. 1) [23].

Environmental situationplays a pivotal role in modulating neurotoxin production. Fluctuations in nutrient concentrationsparticularly nitrogen and phosphorusalong with variations in salinity, pH, and temperature, can induce transcriptional changes in genes encoding key biosynthetic enzymes. These regulatory mechanisms often lead to increased toxin production during algal blooms, suggesting an adaptive ecological function, such as predator deterrence or competitive advantage. Understanding these biochemical pathways is essential not only for elucidating the ecological role of algal neurotoxins but also for developing strategies to mitigate their impacts on marine ecosystems and human health [24–26].

### Challenges posed by neurotoxins and HABs to ecosystems and human health

The presence of HAB-related neurotoxins has far-reaching implications for marine ecosystems, human health, and economic stability. In aquatic environments, neurotoxins disrupt food chains by affecting grazers such as zooplankton and filter-feeding shellfish, leading to bioaccumulation in higher trophic levels. This can result in widespread mortality among marine organisms, including fish and seabirds [27, 28]. From a human health perspective, exposure to neurotoxins via contaminated seafood or aerosolized HAB particles can cause debilitating neurological symptoms. Paralytic shellfish poisoning (PSP), amnesic shellfish poisoning (ASP), and neurotoxic shellfish poisoning (NSP) are severe conditions linked to toxin ingestion. These illnesses range from mild gastrointestinal distress to life-threatening respiratory paralysis or permanent neurological damage. The unpredictability of HAB occurrences complicates regulatory efforts and necessitates continuous monitoring of coastal waters [29].

Beyond their impact on public health, harmful algal blooms (HABs) impose significant socioeconomic burdens. Fisheries suffer substantial losses due to stock contamination, while coastal communities experience declines in tourism revenue. Therefore, Effectively addressing these challenges necessitates interdisciplinary strategies that integrate ecological monitoring, public health interventions, and targeted mitigation efforts to manage bloom triggers [30].

## Nanotechnology in neurotoxin detection

### Development of nano-biosensors for rapid and accurate detection of neurotoxins

The application of nanotechnology in neurotoxin detection has led to the development of highly sensitive nano-biosensors, which significantly enhance the efficiency and accuracy of toxin monitoring. Traditional detection methods, including high-performance liquid chromatography (HPLC) and enzyme-linked immunosorbent assays (ELISA), are effective but often time-consuming, requiring sophisticated laboratory equipment and trained personnel. In contrast, nano-biosensors provide several distinct advantages, including real-time detection capabilities, superior sensitivity, portability, and the potential for multiplexed analysis of multiple neurotoxins simultaneously [31]. These platforms are particularly valuable for routine monitoring of coastal waters, rapid response during HAB events, and point-of-care testing for seafood safety. The working principle of nano-biosensors relies on the integration of nanomaterials with specific bio-recognition elements to convert toxin-analyte interactions into measurable signals. Three main transduction mechanisms are commonly employed: electrochemical, optical, and piezoelectric [32]. Electrochemical biosensors measure current or impedance changes resulting from neurotoxin binding to electrode surfaces modified with nanomaterials such as gold nanoparticles or grapheme [33]. Optical biosensors, including surface plasmon resonance (SPR) and fluorescence-based systems, detect toxin-induced alterations in light absorption or emission. Piezoelectric sensors utilize quartz crystal microbalance technology to measure mass changes upon toxin capture. Each mechanism offers unique advantages depending on the target neurotoxin, sample matrix, and desired detection limit [34].

These biosensors integrate nanomaterials such as gold nanoparticles, graphene-based structures, and quantum dots to facilitate signal amplification and improve molecular interactions with neurotoxins [35]. The high surface-area-to-volume ratio of these nanomaterials provides abundant binding sites for bio-recognition elements, while their unique electronic and optical properties enable signal amplification down to single-molecule detection levels. Additionally, bio-recognition elements such as aptamers, antibodies, and enzymes allow selective binding to neurotoxins, triggering quantifiable responses that ensure precise identification [36]. Among these, aptamers short, single-stranded DNA or RNA sequences have gained particular attention due to their high stability, low cost of synthesis, and excellent reproducibility compared to traditional antibodies.

Recent studies demonstrate that nano‑biosensors can detect marine neurotoxins at extremely low concentrations, with detection limits reported as low as 0.5–5 nM for saxitoxin, 0.1 nM for domoic acid, and even femtomolar levels for botulinum neurotoxin using nanostructured electrodes and aptamer‑based platforms [37, 38]. These values represent a 10–1000‑fold improvement over conventional assays. For context, regulatory safety limits for saxitoxin in seafood are typically set at 80 µg/kg (approximately 160 nM), meaning that current nano-biosensor detection limits are well below regulatory thresholds, offering a substantial safety margin for public health monitoring. The ability to detect neurotoxins at such low concentrations enables early warning of contamination before toxins reach dangerous levels, potentially preventing human poisoning events. Portability is another major advantage, as many nano‑biosensors are now integrated into handheld electrochemical readers, paper‑based microfluidic chips, and smartphone‑connected optical devices weighing less than 200 g, enabling rapid on‑site detection without laboratory infrastructure [39, 40]**.**These portable platforms have been successfully field-tested in various marine environments, demonstrating their practicality for routine monitoring by non-specialized personnel. However, challenges remain in terms of long-term sensor stability, matrix interference from complex environmental samples, and calibration requirements. Current research is actively addressing these limitations through the development of self-calibrating sensor arrays, microfluidic sample pre-treatment modules, and machine learning algorithms for signal interpretation [41–43] (Table 1).

**Table 1:** Fundamental advantages of nano-biosensors in neurotoxin detection. this table comprehensively summarizes the advantages of nano-biosensors in neurotoxin detection, highlighting their role in improving sensitivity, specificity, and efficiency in environmental and biomedical applications

Feature	Scientific advantages	Example neurotoxin & concentration range	Reference
High sensitivity	Enables detection of neurotoxins at trace concentrations, improving early warning capabilities	Saxitoxin (STX): detection limit ~ 0.1–1 ng/mL	[41, 44]
Rapid detection	Reduces analysis time, allowing real-time monitoring in marine environments	Domoic Acid: detection within 10–15 min	[31, 45]
Portability	Compact sensor designs facilitate field deployment for on-site testing	Brevetoxin: portable assays detect < 5 ng/mL	[46, 47]
Cost efficiency	Minimizes reliance on expensive laboratory infrastructure, making monitoring more accessible	General application across HAB toxins	[31, 48, 49]
Selectivity	Utilizes highly specific recognition elements such as aptamers and antibodies to distinguish neurotoxins from other contaminants	Microcystin-LR: aptamer-based selectivity	[49–51]
Miniaturization	Enables the development of small-scale, integrated biosensors for on-site and point-of-care applications	Cylindrospermopsin: chip-based detection	[52–54]
Multiplexing capability	Allows simultaneous detection of multiple neurotoxins, improving diagnostic efficiency	STX + DA multiplex assays	[55, 56]
Real-time monitoring	Provides continuous assessment of neurotoxin levels, facilitating early intervention strategies	HAB toxins monitored continuously	[48, 57]
Eco-friendly design	Reduces chemical waste and environmental impact compared to conventional detection methods	General application	[41, 58, 59]

These advancements are instrumental in environmental monitoring, seafood safety assessment, and public health protection, enabling proactive responses to harmful algal blooms (HABs) [60].

### Integration of marine algae-derived compounds into nanomaterials for enhanced sensitivity

Marine algae produce a diverse array of bioactive compounds that have demonstrated significant potential in improving the functional properties of nanomaterials. Polysaccharides, proteins, and secondary metabolites derived from algae enhance the stability, selectivity, and binding efficiency of nano-biosensors, thereby increasing their overall sensitivity to neurotoxins [61, 62].

The fundamental rationale behind this integration lies in the unique physicochemical properties of algae-derived biomolecules, including their high density of functional groups (e.g., hydroxyl, carboxyl, and sulfate moieties), which facilitate strong interactions with both nanomaterial surfaces and target toxin molecules [63]. These functional groups enable covalent conjugation, electrostatic adsorption, and hydrogen bonding, creating stable and reproducible sensor interfaces [64].

Compounds such as fucoidan (derived from brown algae) and carrageenan (extracted from red algae) exhibit strong biocompatibility and have been successfully incorporated into nanomaterials to optimize toxin detection. These biomolecules facilitate effective interactions between the sensor surface and neurotoxins, leading to improved analytical performance. Fucoidan, a sulfated polysaccharide, contains fucose residues and sulfate ester groups that confer negative charge and high water solubility, making it particularly effective at binding positively charged toxin molecules through electrostatic interactions. Carrageenan, another sulfated polysaccharide, forms helical structures in solution that create binding pockets for small-molecule neurotoxins, enhancing capture efficiency [65, 66].

Several studies have successfully performed this integration. For example, fucoidan from brown algae has been incorporated into biosensor platforms for microcystin-LR detection, achieving limits as low as ~ 0.05 ng/mL [67, 68]. This detection limit is approximately two orders of magnitude lower than the World Health Organization’s provisional guideline value of 1 µg/L for microcystin-LR in drinking water, demonstrating the practical utility of algae-enhanced biosensors for regulatory compliance monitoring [69].

Beyond fucoidan and carrageenan, other algae-derived compounds have also shown promise. Alginate, a polysaccharide from brown algae, has been used to encapsulate nanomaterials in hydrogel matrices, providing three-dimensional sensor architectures with enhanced surface area and analyte accessibility. Phycobiliproteins (e.g., phycoerythrin and phycocyanin) from red and blue-green algae exhibit natural fluorescence properties, enabling their use as optical reporters in fluorescence-based biosensing platforms. Peptides derived from algal proteins have been employed as molecular recognition elements, offering specificity comparable to antibodies but with greater stability and lower production costs [70, 71] (Table 2).

**Table 2:** Functional contributions of algae-derived compounds to nanotechnology-based neurotoxin detection

Algae-derived compound	Role in nano-sensors	Example neurotoxin & detection limit	Ref
Fucoidan (brown algae)	Enhances bio-recognition processes, improving neurotoxin-binding efficiency	Microcystin-LR: detection limit ~ 0.05 ng/mL using fucoidan-modified biosensor	[67, 72]
Carrageenan (red algae)	Strengthens sensor stability and supports surface modifications for increased accuracy	κ-Carrageenan biosensor applied for toxin-binding stability; detection in μg/mL range	[68, 73]
Marine-derived peptides	Facilitates molecular interactions, boosting specificity in toxin identification	Tetrodotoxin (TTX): peptide-based biosensor detection limit ~ 1 ng/mL	[74–76]
Algal polysaccharides	Improves sensor adhesion, enhancing overall sensitivity and signal strength	General HAB toxins: polysaccharide coatings improve adhesion and sensitivity	[33, 77]
Alginate (brown algae)	Provides structural integrity to nanoparticles, improving biocompatibility and controlled release	Microcystin-LR: alginate nanoparticle biosensor detection limit ~ 0.1 ng/mL	[77, 78]
Phycobiliproteins (red algae)	Enhances fluorescence-based biosensing, improving signal detection for neurotoxin identification	Fluorescence biosensor for saxitoxin (STX): detection limit ~ 0.5 ng/mL using phycobiliproteins	[79–81]
Sulfated polysaccharides	Modulates nanoparticle surface charge, optimizing interactions with neurotoxic molecules	Neuroprotective biosensor coatings for cyanotoxins; detection in ng/mL range	[8, 82–84]
Carotenoids (green and red algae)	Acts as antioxidant stabilizers, preventing oxidative degradation of biosensor components	Botulinum neurotoxin A: portable biosensor assay detection limit ~ 1 ng/mL	[85–87]

highlighting that integration has already been demonstrated across multiple toxin classes. These examples confirm that algae‑derived biomolecules are not only theoretically promising but have been practically incorporated into nanomaterials, resulting in measurable improvements in biosensor performance. Such advancements hold promising applications for environmental monitoring, food safety, and biomedical research, contributing to innovative strategies for mitigating HAB‑related risks [88].

## Nano-based mitigation strategies for harmful algal blooms (HABs)

### Use of nanotechnology in environmental monitoring and HAB management

The integration of nanotechnology in environmental monitoring and HAB management has emerged as a transformative approach to detecting, controlling, and mitigating the adverse effects of harmful algal blooms. Traditional monitoring techniques, such as satellite imaging and water sampling, provide valuable data but often lack real-time sensitivity and precision. Nanotechnology offers innovative solutions by enhancing detection accuracy, enabling early intervention, and minimizing ecological disruptions [89, 90].

Nano-sensors, which utilize nanomaterials such as carbon nanotubes, quantum dots, and metal oxide nanoparticles, provide highly sensitive platforms for monitoring HAB-related parameters. These sensors can detect minute changes in water chemistry, including nutrient concentrations and algal toxin levels, allowing for timely responses to bloom developments. Furthermore, nano-based fluorescence and electrochemical sensors facilitate rapid identification of neurotoxins, improving risk assessment and management strategies for affected ecosystems [89, 91, 92].

Another critical application of nanotechnology is the development of nano-enabled remediation systems. By engineering nanostructures capable of binding and neutralizing algal toxins, researchers aim to mitigate the ecological and health hazards posed by HABs. Functionalized nanoparticles with high affinity for neurotoxins can remove contaminants from aquatic systems, reducing their bioavailability and toxic impact [93, 94] (Table 3).

**Table 3:** Applications of nanotechnology in environmental monitoring and HAB management

Nanotechnology application	Function in HAB monitoring and management	Example neurotoxin & detection limit	References
Nano-sensors	Detects environmental changes and toxin presence with high sensitivity, enabling early intervention strategies	Saxitoxin (STX): aptamer-based nanosensor detection limit ~ 0.1 ng/mL	[44, 95]
Quantum dot fluorescence sensors	Provides real-time visualization of HAB-associated toxins, improving detection accuracy in aquatic environments	Domoic Acid: quantum dot biosensor detection limit ~ 0.5 ng/mL	[96–99]
Electrochemical nano-sensors	Enables precise quantification of neurotoxin concentrations, facilitating rapid risk assessment and mitigation	Microcystin-LR: electrochemical aptasensor detection limit ~ 0.05 ng/mL	[49, 100, 101]
Nano-Enabled Remediation	Neutralizes and removes algal toxins from aquatic ecosystems, reducing bioavailability and toxic impact	Cylindrospermopsin: photocatalytic degradation using TiO₂ nanoparticles	[54, 102]
Nanoparticle-based adsorbents	Utilizes functionalized nanoparticles to capture and degrade algal toxins, preventing their accumulation in marine food chains	Microcystin-LR: adsorption using graphene oxide nanoparticles, detection limit ~ 0.1 ng/mL	[78, 103]
Magnetic nanoparticles	Facilitates toxin removal through magnetic separation techniques, enhancing water purification processes	Brevetoxin: removal efficiency 90% using Fe₃O₄ nanoparticles	[89, 104]
Nano-coatings for sensors	Enhances sensor durability and sensitivity, ensuring long-term monitoring of HAB-related parameters	Microcystin-LR: nano-coating improved stability and detection limit ~ 0.05 ng/mL	[57, 105]
DNA-based nano-biosensors	Detects HAB species at the genetic level, enabling early identification and predictive modeling of bloom events	Karenia brevis (brevetoxin-producing species): DNA biosensor detection limit ~ 10 copies/µL	[106]
Nano-photocatalysts	Degrades algal toxins through photocatalytic reactions, reducing their persistence in aquatic environments	Microcystin-LR: photocatalytic degradation using TiO₂ nanomaterials	[107]
Smart nanomaterials for HAB control	Develops responsive nanomaterials that inhibit algal proliferation, preventing bloom formation without harming non-target organisms	Cylindrospermopsisraciborskii: smart nanomaterials suppress bloom formation	[84, 108, 109]

These advancements underscore the potential of nanotechnology in proactive HAB management, reducing ecological damage while safeguarding public health [5, 89].

### Novel nanomaterials for neutralizing neurotoxins in aquatic systems

Beyond monitoring capabilities, nanotechnology plays a crucial role in the direct mitigation of neurotoxin contamination. The development of engineered nanomaterials capable of neutralizing harmful algal toxins presents a promising strategy for minimizing their impact on marine ecosystems and human health [110].

Nanoparticles with surface modifications designed for toxin adsorption and degradation offer targeted solutions for toxin removal. For instance, metal-based nanostructures, such as titanium dioxide and silver nanoparticles, have been explored for their ability to degrade neurotoxic compounds through photocatalytic and oxidative processes [111, 112]. Additionally, functionalized carbon-based nanomaterials, including graphene and activated carbon nanoparticles, exhibit strong adsorption properties, effectively capturing and sequestering algal toxins from water bodies [113]. The following table (Table 4) outlines key nanomaterials utilized in neurotoxin neutralization, including representative toxins and detection/mitigation ranges reported in peer-reviewed studies:

**Table 4:** The following table outlines key nanomaterials utilized in neurotoxin neutralization:

Nanomaterial	Mechanism of neurotoxin mitigation	Example neurotoxin & detection/mitigation range	Ref
Titanium dioxide nanoparticles	Photocatalytic degradation of neurotoxins under light exposure	Microcystin-LR: photocatalytic degradation efficiency 90% under UV light	[47, 114]
Silver nanoparticles	Oxidative neutralization of algal toxins, reducing their toxicity	Saxitoxin: oxidative degradation, detection limit ~ 1 ng/mL	[115, 116]
Graphene-based nanostructures	Adsorption and sequestration of neurotoxins, preventing bioaccumulation	Microcystin-LR: adsorption capacity ~ 50 mg/g	[77]
Activated carbon nanoparticles	Efficient binding and filtration of toxins from aquatic environments	Domoic Acid: removal efficiency 85% in water samples	[117]

**Fig. 2:**
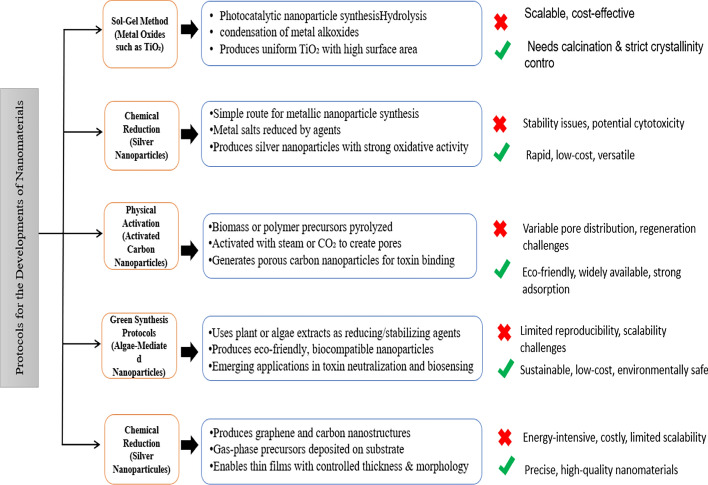
Flowchart of protocols for nanomaterial development in neurotoxin mitigation. The schematic illustrates five major synthesis routes sol‑gel processing, chemical reduction, chemical vapor deposition, physical activation, and green synthesis. Each protocol is shown with its key steps and representative outcomes, leading to nanomaterials such as titanium dioxide, silver nanoparticles, graphene‑based structures, activated carbon, and algae mediated particles. These methods collectively highlight how synthesis strategies influence adsorption, degradation, and sequestration properties critical for neutralizing aquatic neurotoxins. In the flowchart, the green tick (✔) indicates the advantages of each method, while the red cross (✘) denotes the disadvantages, providing a clear comparative view of their strengths and limitations

To visually clarify the synthesis strategies discussed, a detailed flowchart (Fig. 2) has been included to illustrate the core protocols used in nanomaterial development for neurotoxin mitigation. These schematic outlines five major approaches sol–gel synthesis, chemical reduction, chemical vapor deposition, physical activation, and green synthesis, each broken down into key steps and outcomes. These protocols lead to the formation of functional nanomaterials such as titanium dioxide, silver nanoparticles, graphene-based structures, activated carbon, and algae-mediated particles, all tailored for adsorption, degradation, or sequestration of aquatic neurotoxins. By mapping the progression from raw inputs to optimized nanostructures, the flowchart complements figuer 2 and enhances the reader’s understanding of how synthesis methodology directly influences performance in environmental applications.

The application of these nanomaterials represents an innovative approach to reducing neurotoxin concentrations in marine ecosystems, addressing both environmental and public health concerns. Further research into the optimization of nanomaterial properties and ecological safety will enhance their effectiveness in real-world HAB mitigation efforts [118, 119].

## Therapeutic and pharmacological potential of marine neurotoxins

### Exploration of neurotoxins as tools for neuroscience research

Marine neurotoxins have emerged as critical tools in neuroscience research due to their ability to selectively target ion channels and neurotransmitter systems. These naturally occurring compounds, derived from marine algae and associated organisms, offer unique insights into neuronal function and have played a pivotal role in advancing the understanding of neurophysiology and neurodegenerative diseases [120, 121]. Historically, the use of neurotoxins in neuroscience dates back several decades, with tetrodotoxin isolated from pufferfish serving as one of the first molecular tools to dissect sodium channel function. Since then, marine algae-derived neurotoxins have become indispensable probes for investigating fundamental questions in neurobiology, ranging from synaptic transmission to neural network dynamics [122, 123].

From a mechanistic perspective, the utility of marine neurotoxins in neuroscience research stems from their exceptional binding affinity and specificity toward distinct classes of ion channels and receptors [123]. Unlike synthetic compounds that often exhibit off-target effects, these naturally evolved toxins have been refined through millions of years of evolutionary selection to interact with precise molecular targets. This specificity enables researchers to isolate and study individual components of the neuronal signaling apparatus without confounding variables introduced by non-selective pharmacological agents. The following subsections detail the specific contributions of the most widely studied marine neurotoxins to neuroscience research [124].

One of the primary applications of marine neurotoxins in neuroscience is their ability to modulate voltage-gated ion channels, which are essential for neuronal excitability and signal transmission. Saxitoxin and tetrodotoxin, both potent sodium channel blockers, have been extensively utilized in electrophysiological studies to investigate action potential propagation and nerve impulse regulation. These toxins bind to the pore region of voltage-gated sodium channels with nanomolar affinity, physically occluding sodium ion flow and thereby blocking depolarization. Different subtypes of sodium channels (Nav1.1 through Nav1.9) exhibit varying sensitivities to saxitoxin and tetrodotoxin, allowing researchers to determine which channel subtypes are expressed in specific neuronal populations and how they contribute to distinct physiological functions. Their specificity allows researchers to delineate the role of sodium channels in various physiological processes and neurological disorders, including epilepsy, chronic pain, and neurodegeneration [125, 126]. For example, the differential sensitivity of Nav1.8 channels to tetrodotoxin has enabled the identification of this subtype as a key mediator of inflammatory pain, leading to the development of selective Nav1.8 inhibitors now in clinical trials for pain management [127].

Domoic acid, another well-characterized marine neurotoxin, has been instrumental in studying excitotoxicity, a phenomenon linked to excessive glutamate receptor activation. This toxin mimics glutamate, leading to overstimulation of neurons and eventual cell death, closely mirroring pathological processes observed in Alzheimer’s disease and other forms of dementia. Specifically, domoic acid acts as a potent agonist of kainate receptors, a subclass of ionotropic glutamate receptors [128]. At nanomolar concentrations, domoic acid induces sustained receptor activation, resulting in prolonged calcium influx, mitochondrial dysfunction, generation of reactive oxygen species, and ultimately apoptotic or necrotic cell death. This molecular cascade recapitulates key features of excitotoxic neurodegeneration observed in conditions such as stroke, traumatic brain injury, and amyotrophic lateral sclerosis (ALS) [128, 129].

By using domoic acid-induced neurotoxicity as a model, researchers have explored potential neuroprotective strategies aimed at mitigating glutamate-induced neuronal damage [130, 131].

Preclinical studies utilizing domoic acid exposure have identified several promising neuroprotective agents, including calcium chelators, antioxidant compounds, and glutamate receptor antagonists, some of which have advanced to clinical evaluation for neurodegenerative diseases [132]. Beyond their use in basic research, marine neurotoxins have provided valuable insights into drug discovery and therapeutic intervention. Their highly specific interactions with neural receptors make them viable candidates for pharmacological refinement, particularly in the development of treatments for pain management, neuroinflammation, and neuromuscular disorders. In particular, the structure–activity relationships of saxitoxin and tetrodotoxin have guided medicinal chemistry efforts to develop non-addictive analgesics. By chemically modifying these neurotoxins to preferentially target peripheral sodium channels while reducing central nervous system penetration, researchers have produced analogs that maintain analgesic efficacy without the cardiorespiratory toxicity associated with the parent compounds. Several such derivatives are currently undergoing preclinical evaluation as long-acting local anesthetics for postoperative pain management [133].

The precise molecular mechanisms elucidated through neurotoxin studies continue to inform advancements in neuropharmacology, shaping innovative approaches to neurological therapeutics [134–136]. Furthermore, neurotoxin-based molecular tools have facilitated the discovery of previously unknown ion channel subtypes and the characterization of their distribution patterns across the central and peripheral nervous systems, knowledge that is essential for developing subtype-selective therapeutic agents [137].

In addition, several reports highlight the development of different types of nano-sensors designed to detect and analyze marine neurotoxins with high sensitivity. These include electrochemical nano-sensors (using nanostructured electrodes for saxitoxin detection), optical nano-sensors such as SPR and SERS platforms (employing gold nanoparticles for domoic acid analysis), carbon-based sensors (graphene and CNT systems for microcystin-LR), and algae-derived compound-integrated sensors (fucoidan, carrageenan, and peptides for enhanced biocompatibility) [95]. Each sensor type follows distinct development strategies but also faces practical limitations: electrochemical sensors require expensive electrode modifications, optical sensors depend on costly instrumentation, carbon-based sensors struggle with reproducibility at scale, and algae-derived sensors encounter stability and storage challenges [138]. A critical analysis reveals that while electrochemical sensors offer the advantage of portability and rapid response, their sensitivity is often compromised in complex biological matrices such as serum or cerebrospinal fluid [139]. Optical sensors provide superior sensitivity but require sophisticated infrastructure, limiting their deployment in resource-limited laboratory settings. Carbon-based sensors, despite their excellent electrical properties, suffer from batch-to-batch variability in nanomaterial synthesis, affecting assay reproducibility. Algae-derived sensors, while promising from a biocompatibility standpoint, face challenges related to the seasonal and geographical variability of algal biomass, which impacts the consistency of extracted biomolecules. An in-depth analysis of these nano-sensors demonstrates both their promise in advancing neurotoxin research and the practical limitations that must be addressed for real-world applications [140–145].

### Utilization of nanocarriers for targeted delivery of marine neurotoxins in drug development

The pharmacological potential of marine neurotoxins has garnered significant attention in drug development, particularly in the field of targeted drug delivery. While these compounds exhibit strong bioactivity, their potent effects necessitate controlled administration to minimize systemic toxicity. Nanotechnology presents a promising solution by enabling precise transport and release of neurotoxin-based therapeutics at specific biological targets, ensuring efficacy while mitigating adverse effects [146, 147].

To achieve selective delivery, modern nanocarrier systems incorporate a variety of targeting strategies. One widely used approach involves surface functionalization with targeting ligandssuch as peptides, antibodies, aptamers, folate, or transferring that bind specifically to receptors overexpressed on neuronal or glial cells [148]. These ligands guide nanocarriers toward diseased neural tissues, enhancing accumulation at the intended site while reducing systemic exposure. Biomimetic modifications represent another powerful strategy: coating nanoparticles with cell‑derived membranes (e.g., neuronal, macrophage, or stem‑cell membranes) enables immune evasion, prolonged circulation, and natural homing toward inflamed or degenerating neural regions [149]. Additionally, cell‑penetrating peptides (CPPs) and receptor‑mediated transcytosis motifs facilitate efficient crossing of biological barriers such as the blood–brain barrier, enabling neurotoxins to reach deep neural targets with high precision [150]. Together, these targeting mechanisms significantly improve the therapeutic index of marine neurotoxin‑based formulations by enhancing specificity, reducing off‑target toxicity, and enabling controlled pharmacological action [151].

Nanocarriers such as liposomes, polymeric nanoparticles, and lipid-based vesicles have been developed to encapsulate marine neurotoxins, enhancing their stability, bioavailability, and therapeutic specificity. Liposomal formulations, for instance, provide a protective lipid bilayer that shields neurotoxins from enzymatic degradation and premature clearance, allowing for sustained drug release. Polymeric nanoparticles offer controlled delivery mechanisms that regulate toxin release dynamics, ensuring prolonged pharmacological action while preventing abrupt fluctuations in bioavailability [152–154].

A particularly compelling area of research is the development of nano-enabled analgesics based on sodium channel-blocking neurotoxins such as saxitoxin and tetrodotoxin. Encapsulation of these compounds within biodegradable nanoparticles has demonstrated potential for localized pain relief, presenting an alternative to opioid-based medications. By targeting peripheral nerves while avoiding systemic neurotoxicity, these formulations offer a promising direction for non-addictive, long-duration pain management strategies [125, 155].

In addition to neurotoxin encapsulation, nanocarriers facilitate receptor-specific targeting, allowing for precision medicine approaches in neurology. Functionalized nanoparticles can be engineered to recognize and bind to neural receptors associated with neurodegenerative diseases, improving drug efficacy while reducing off-target effects. Continued advancements in nanocarrier technology are expected to refine delivery systems further, enhancing the pharmacokinetic properties of marine neurotoxin-based therapeutics [156, 157].

The integration of marine neurotoxins with nanotechnology marks a significant advancement in neuroscience and neuropharmacology, providing innovative solutions for neurological disorders and precision therapeutics. Future research efforts should focus on optimizing nanocarrier formulations and improving biocompatibility to fully harness the therapeutic potential of these marine-derived compounds [158].

## Marine algae in nanotheranostics

### Role of marine algae-derived compounds in synthesizing nanoparticles for imaging and therapy

Marine algae represent a valuable source of bioactive compounds with significant potential in the synthesis of nanoparticles for biomedical applications. These naturally derived biomolecules, including polysaccharides, proteins, and secondary metabolites, offer advantages such as biocompatibility, stability, and enhanced functional properties, making them ideal candidates for nanoparticle formulation [63, 159]. The growing interest in green nanotechnology has positioned marine algae as particularly attractive biofactories for nanomaterial synthesis, as they eliminate the need for toxic chemical reducing and capping agents traditionally used in conventional nanoparticle production methods [160]. Unlike chemically synthesized nanoparticles, which often require hazardous reagents such as sodium borohydride or hydrazine and generate environmental waste, algae-mediated synthesis offers a sustainable, cost-effective, and environmentally benign alternative. Furthermore, the inherent bioactivity of algae-derived compounds can impart additional therapeutic or targeting functionalities to the resulting nanoparticles, creating multifunctional platforms not achievable through purely synthetic approaches [3, 161].

From a mechanistic standpoint, the synthesis of nanoparticles using marine algae-derived compounds proceeds through three distinct phases: activation, growth, and termination. During the activation phase, bioactive molecules (primarily polysaccharides, peptides, and phenolic compounds) reduce metal ions (e.g., Ag⁺, Au3⁺, Fe2⁺/Fe3⁺) to their zero-valent metallic states through electron transfer reactions mediated by functional groups such as hydroxyl (-OH), carbonyl (C=O), and amine (-NH_2_) moieties. Simultaneously, these same biomolecules serve as capping agents, adsorbing onto the nascent nanoparticle surface and preventing uncontrolled aggregation. In the growth phase, additional metal ions diffuse to the surface of the nucleated particles, where further reduction occurs, leading to particle size enlargement. The termination phase occurs when the capping agents fully passivate the nanoparticle surface, halting further growth. The size, shape, and crystallinity of the resulting nanoparticles are determined by the specific algal species used, the concentration of precursor metal ions, pH, temperature, and reaction time, allowing for controlled synthesis of nanoparticles with desired physicochemical properties [3, 63, 159].

In imaging applications, algae-derived nanoparticles are used across multiple environments, including in vitro cellular imaging, in vivo preclinical imaging, and environmental biosensing. Their imaging performance is based on several principles: (i) optical imaging, where algal pigments or metal enhanced nanoparticles generate fluorescence or luminescence signals; (ii) magnetic resonance imaging (MRI), where algae‑mediated synthesis of iron oxide nanoparticles provides strong T1/T2 contrast; (iii) photoacoustic imaging, in which nanoparticles absorb near infrared light and convert it into acoustic waves for deep tissue visualization; and (iv) computed tomography (CT) or X-ray imaging, where high atomic number nanoparticles (e.g., Au, Ag) synthesized using algal extracts enhance radiographic contrast. These imaging mechanisms enable improved signal intensity, deeper tissue penetration, and enhanced spatial resolution, supporting applications in tumor localization, vascular imaging, and real‑time tracking of therapeutic delivery [162–164]. A comparative evaluation reveals that algae-synthesized imaging nanoparticles often outperform chemically synthesized counterparts in terms of biocompatibility and reduced immunogenicity. For instance, iron oxide nanoparticles synthesized using Sargassum extract exhibit comparable or superior T2 relaxivity to conventionally prepared agents while demonstrating significantly lower cytotoxicity in neuronal cell lines. However, challenges remain in achieving consistent size distribution and crystallinity across different algal batches, factors that critically influence imaging performance reproducibility. Current research is addressing these limitations through optimization of extraction protocols, standardization of reaction parameters, and development of post-synthetic purification methods [165–167].

One of the primary applications of marine algae in nanomedicine is their role in facilitating the synthesis of metal-based and polymeric nanoparticles for imaging and therapeutic interventions. Algal polysaccharides, such as fucoidan and carrageenan, act as stabilizing and capping agents in nanoparticle synthesis, ensuring controlled size distribution and improved biocompatibility. The ability of these biomolecules to enhance nanoparticle stability and reduce toxicity makes them particularly suitable for targeted drug delivery and contrast-enhanced imaging techniques in biomedical diagnostics [168–170]. Fucoidan-based nanoparticles have demonstrated selective uptake by macrophages and cancer cells due to the interaction of fucoidan with P-selectin and scavenger receptors, which are overexpressed on activated endothelial cells and various tumor types. This receptor-mediated targeting capability enables passive and active accumulation of fucoidan-coated nanoparticles at diseased sites, reducing off-target distribution and enhancing therapeutic indices. Carrageenan, with its negative surface charge, facilitates electrostatic interactions with positively charged cell membranes and has been successfully incorporated into mucoadhesive drug delivery systems for ophthalmic and oral administration. Notably, carrageenan-based nanoparticles have shown enhanced permeability and retention (EPR) effect-mediated tumor accumulation in preclinical models, achieving up to 3.5-fold higher tumor drug concentrations compared to uncoated controls [171, 172].

In addition to their structural function, marine algae-derived compounds exhibit intrinsic therapeutic properties that further expand their utility in nanotheranostics. For example, bioactive molecules extracted from *Ulva* and *Sargassum* species possess antioxidant and anti-inflammatory effects, contributing to nanoparticle-mediated therapeutic strategies. These compounds have been explored for their potential in cancer therapy, where nanoparticle formulations improve drug targeting while minimizing adverse effects on healthy tissues[63, 173, 174]. Specifically, ulvan (a sulfated polysaccharide from Ulva) exhibits immunomodulatory activity through activation of Toll-like receptor 4 (TLR4) and promotes macrophage polarization toward the antitumor M1 phenotype. Sargassum-derived fucoidan has been shown to induce apoptosis in various cancer cell lines via caspase-3 and caspase-9 activation while simultaneously inhibiting angiogenesis through downregulation of vascular endothelial growth factor (VEGF). When incorporated into nanoparticle platforms, these intrinsic therapeutic effects synergize with encapsulated chemotherapeutic agents, enabling combination therapy approaches that may overcome drug resistance mechanisms. Furthermore, the antioxidant properties of these compounds (demonstrated by DPPH radical scavenging and ferric reducing antioxidant power (FRAP) assays) can mitigate the oxidative stress induced by certain chemotherapeutic agents, potentially reducing treatment-related toxicities in normal tissues [175, 176].

Despite the considerable promise of algae-mediated nanoparticle synthesis, several challenges impede clinical translation. Scale-up from laboratory-scale batch synthesis (milligrams to grams) to industrial-scale production (kilograms) remains technically challenging, as reaction conditions optimized for small volumes often fail to yield consistent nanoparticle properties at larger scales[177]. Regulatory approval pathways for algae-derived nanomaterials are not yet well-established, as existing frameworks were designed for synthetically produced nanoparticles and do not fully accommodate the inherent batch-to-batch variability of biologically sourced materials nt batch-to-batch variability of biologically sourced materials [178]. Furthermore, comprehensive toxicity studies evaluating long-term biodistribution, degradation, and clearance of algae-synthesized nanoparticles are currently lacking. Addressing these knowledge gaps requires interdisciplinary collaboration among marine biologists, nanochemists, materials scientists, and regulatory toxicologists. Future research priorities should include: (i) development of continuous flow synthesis systems for reproducible large-scale production; (ii) establishment of standardized quality control metrics specific to algae-derived nanomaterials; (iii) comprehensive in vivo toxicokinetic and long-term safety studies in relevant animal models; and (iv) investigation of the fate of algal biomolecules within biological systems to identify potential immunogenic or off-target effects [179].

The integration of marine algae-derived components into nanoparticle synthesis not only enhances the functional properties of nanomaterials but also aligns with the growing demand for environmentally sustainable and biologically compatible alternatives in biomedical applications [180].

### Application of marine neurotoxins in theranostic platforms combining diagnostics and treatment

The unique biochemical properties of marine neurotoxins have positioned them as promising candidates for theranostic applications, where diagnostic and therapeutic functions are combined within a single platform. These potent bioactive compounds, traditionally associated with toxicological concerns, are now being explored for their potential in targeted treatment strategies for neurological disorders, pain management, and cancer therapy [146, 181].

For example, One emerging application involves the use of neurotoxin-based nanoparticles for precision imaging of neurodegenerative conditions [182]. Certain neurotoxins, such as saxitoxin and brevetoxins, exhibit strong affinities for neuronal receptors, enabling selective interaction with diseased neural tissues. When conjugated with nanoparticles designed for imaging, these neurotoxins enhance diagnostic precision, facilitating early detection of neurological disorders such as Alzheimer's disease and multiple sclerosis [182, 183].

The development of theranostic platforms incorporating marine neurotoxins underscores the growing interdisciplinary focus on nanomedicine, neuropharmacology, and toxin-based therapeutics. Continued research into optimizing these systems will be essential for translating neurotoxin-based nanotechnology into clinical applications while ensuring safety and efficacy in human health interventions [183].

## Future perspectives

### Potential research directions at the intersection of marine algae and nanotechnology

The convergence of marine algae and nanotechnology presents an emerging frontier with significant implications for biomedicine, environmental sustainability, and material sciences. While substantial progress has been made in harnessing marine algae-derived compounds for nanomaterial synthesis, several areas require further exploration to maximize their potential applications [180].

One key direction involves refining algae-based nanocarriers for targeted drug delivery. The development of nanoparticles functionalized with marine algae-derived biomolecules may improve drug bioavailability, reduce systemic toxicity, and enhance therapeutic efficacy. By integrating biologically active compounds from algae, researchers can explore their role in modulating nanocarrier interactions with diseased tissues, potentially enabling site-specific therapeutic interventions for neurodegenerative diseases, cancer, and antimicrobial treatments [184, 185].

The environmental applications of marine algae‑derived nanomaterials are another promising avenue. Algae‑based nanoparticles have demonstrated potential in water purification and pollutant remediation due to their natural adsorptive properties. Further studies should investigate the optimization of these materials to enhance their efficiency in removing heavy metals, organic pollutants, and harmful algal toxins from aquatic ecosystems. Understanding how structural modifications influence their interaction with contaminants will be essential for their large-scale deployment in sustainable environmental management [63, 186].

Additionally, integrating marine neurotoxins into nanotechnology platforms offers new possibilities in biomedical imaging and theranostics. Some neurotoxins exhibit specificity for neuronal receptors, making them potential candidates for precision diagnostics in neurological disorders. Future research should focus on engineering nanoparticle formulations that stabilize and regulate neurotoxin activity, ensuring their controlled use in targeted imaging systems and therapeutics [187, 188].

While these research directions provide exciting opportunities, interdisciplinary collaboration will be fundamental to translating laboratory findings into practical applications. Advancements in nanobiotechnology, materials science, and marine biology must intersect to refine methodologies and ensure the safe and effective use of algae-derived nanomaterials.

To guide future research, a comparative overview of reported studies has been provided in Table 5. This table highlights the advantages and disadvantages of different approaches, including HAB monitoring strategies, neurotoxin pharmacology, nano‑biosensor development, and the integration of algae‑derived compounds into nanomaterials. By synthesizing detection limits, performance outcomes, and practical challenges, the table not only summarizes the current state of knowledge but also underscores where innovation is most needed. Linking these insights with the proposed research directions ensures that future work can build on existing strengths while addressing limitations, ultimately accelerating the translation of marine algae–nanotechnology applications into real‑world solutions.

**Table 5:** Comparative overview of reported studies and their advantages and disadvantages

Focus Area	Example study / Target	Reported data (Detection limit / Impact)	Advantages	Disadvantages	References
HABs monitoring	Chlorophyll-a thresholds for bloom detection	10 µg/L chlorophyll-a; cell density 10^6^ cells/L	Simple proxy, widely used	Non-specific, may miss toxin presence	[189, 190]
Economic impact of HABs	Fisheries & aquaculture losses	Billions annually in closures & healthcare costs	Highlights societal relevance	Data varies regionally, hard to generalize	[191, 192]
Neurotoxin pharmacology	Saxitoxin, brevetoxin, domoic acid	STX: sodium channel blocker; DA: glutamate agonist	High specificity, research tools	Severe toxicity, limited safe use	[146, 193]
Nano-biosensors	STX, DA, Botulinum toxin	STX: 0.5–5 nM; DA: 0.1 nM; BoNT: femtomolar	10–1000 × more sensitive than ELISA/HPLC	Device cost, stability issues	[194]
Integration of algae-derived compounds	Fucoidan, carrageenan, alginate, peptides	MC-LR: 0.05 ng/mL (fucoidan); TTX: ~ 1 ng/mL (peptides); STX: ~ 0.5 ng/mL (phycobiliproteins)	Biocompatibility, improved binding, stability	Extraction complexity, variability	[195, 196]
Theranostics& Nanomedicine	Neurotoxin-loaded nanocarriers	Controlled release, imaging, precision targeting	Enables dual therapy/diagnostics	Ethical concerns, toxicity risk	[197, 198]

### Strategies for overcoming ethical and environmental challenges

Despite the transformative potential of marine algae-based nanotechnology, several ethical and environmental considerations must be addressed to ensure responsible implementation. A major concern is the sustainability of algae harvesting, particularly in fragile marine ecosystems. The large-scale extraction of bioactive compounds for nanomaterial production could disrupt ecological balance and threaten biodiversity. Therefore, Developing controlled cultivation strategies, such as microalgae bioreactors and closed-loop aquaculture systems, may mitigate these risks by reducing dependence on wild algae populations while ensuring consistent yields [199, 200].

Another ethical challenge pertains to the safe deployment of marine neurotoxins in biomedical applications. While these compounds hold promise for targeted drug delivery and theranostic platforms, their inherent toxicity necessitates stringent regulatory oversight [201]. Consequently, Future studies should prioritize the development of biocompatible nanocarriers that enable controlled neurotoxin release while minimizing unintended biological interactions. In addition, Establishing standardized toxicity assessments and conducting long-term safety evaluations will be crucial in determining their suitability for clinical applications [202, 203].

Furthermore, concerns regarding the environmental impact of nanotechnology must be carefully considered. The potential accumulation of synthetic nanoparticles in ecosystems raises questions about their long-term effects on marine life and human health [204]. However, The design of biodegradable and eco-friendly nanomaterials derived from marine algae offers a viable solution to minimize these risks. Researchers should focus on engineering nanoparticles that degrade naturally without leaving harmful residues, ensuring their safe integration into medical, industrial, and environmental processes [205].

The ethical and regulatory landscape surrounding algae-based nanotechnology must evolve alongside scientific advancements. Therefore, Collaborative frameworks involving researchers, policymakers, and industry stakeholders will be necessary to establish guidelines that balance innovation with sustainability [206]. Furthermore, Emphasizing transparency, ecological responsibility, and equitable access to emerging technologies will ultimately shape the future of marine algae-derived nanomaterials as a viable solution for global health and environmental challenges [207].

## Conclusion

This review has synthesized current knowledge on harmful algal blooms (HABs), marine algae‑derived neurotoxins, and their integration into nanotechnology platforms. HABs remain a pressing ecological and economic challenge, with cell densities exceeding 10^6 cells/L and chlorophyll-a thresholds above 10 µg/L serving as critical indicators of bloom events. Reported studies highlight their severe impacts on marine ecosystems, fisheries, and public health, with annual economic losses reaching billions worldwide.

Marine algae-derived neurotoxins such as saxitoxin, brevetoxin, and domoic acid, while hazardous in uncontrolled contexts, have demonstrated unique biochemical properties that make them valuable tools in neuroscience and pharmacology. Their ability to selectively modulate ion channels and receptors has opened new avenues for biomedical research, particularly in modeling neurological disorders.

Nanotechnology provides a powerful framework for utilizing marine algae‑derived compounds in a safe and controlled manner. Recent advances in nano‑biosensors have pushed detection capabilities to unprecedented levels, with sensitivities reaching nanomolar and even femtomolar ranges representing improvements of one to three orders of magnitude compared to conventional assays. Studies have further shown that biomolecules such as fucoidan, carrageenan, alginate, peptides, and phycobiliproteins can be incorporated into nanomaterials to enhance sensor stability, biocompatibility, and analytical precision. Table 5 offers a comparative overview of these reported studies, outlining both their strengths and limitations to provide a consolidated perspective on current progress.

From our analysis, the intersection of marine biology and nanotechnology emerges as a promising frontier with broad implications. While notable strides have been made in biosensing, theranostics, and environmental monitoring, persistent challenges remainparticularly in terms of scalability, reproducibility, and long‑term safety. Moving forward, research should prioritize the refinement of algae-based nanocarriers for targeted drug delivery, the optimization of algae‑derived nanomaterials for pollutant remediation, and the design of neurotoxin‑integrated nanoplatforms for precision diagnostics. Therefore, achieving these goals will need close teamwork across disciplines, ensuring that laboratory innovations can be translated into practical solutions for both ecological sustainability and biomedical advancement.

## Data Availability

No datasets were generated or analysed during the current study.

## References

[1] Wong WW, Greening C, Shelley G, Lappan R, Leung PM, Kessler A, et al. Effects of drift algae accumulation and nitrate loading on nitrogen cycling in a eutrophic coastal sediment. Sci Total Environ. 2021;790:147749. 34091344 10.1016/j.scitotenv.2021.147749

[2] Franco-Morgado M, Amador-Espejo GG, Pérez-Cortés M, Gutiérrez-Uribe JA. Microalgae and cyanobacteria polysaccharides: important link for nutrient recycling and revalorization of agro-industrial wastewater. Appl Food Res. 2023;3(1):100296.

[3] de Almeida PDO, Bozorgzadeh SA, Martins IJF, Golbashirzadeh M. Marine algae-derived nanoparticles (MADNs): green synthesis, characterization, and therapeutic applications. Discover Appl Sci. 2025;7(5):1–25.

[4] Anderson DM, Fensin E, Gobler CJ, Hoeglund AE, Hubbard KA, Kulis DM, et al. Marine harmful algal blooms (HABs) in the United States: History, current status and future trends. Harmful Algae. 2021;102:101975. 33875183 10.1016/j.hal.2021.101975 PMC8058451

[5] Lan J, Liu P, Hu X, Zhu S. Harmful algal blooms in eutrophic marine environments: causes, monitoring, and treatment. Water. 2024;16(17):2525.

[6] Paranthaman S, Palraj P. Bioactive compounds of algae: potential neuroprotective agents in neurodegenerative disorders. Neuroprotective effects of phytochemicals in brain ageing: Springer; 2024:257-88.

[7] Barbalace MC, Malaguti M, Giusti L, Lucacchini A, Hrelia S, Angeloni C. Anti-inflammatory activities of marine algae in neurodegenerative diseases. Int J Mol Sci. 2019;20(12):3061. 31234555 10.3390/ijms20123061 PMC6628294

[8] Olasehinde TA, Olaniran AO, Okoh AI. Sulfated polysaccharides of some seaweeds exhibit neuroprotection via mitigation of oxidative stress, cholinergic dysfunction and inhibition of Zn–induced neuronal damage in HT-22 cells. BMC complementary medicine and therapies. 2020;20:1–10. 32799855 10.1186/s12906-020-03047-7 PMC7429825

[9] Figueiredo J, Oliveira T, Ferreira V, Sushkova A, Silva S, Carneiro D, et al. Toxicity of innovative anti-fouling nano-based solutions to marine species. Environ Sci Nano. 2019;6(5):1418–29.

[10] Montecucco C, Schiavo G, Dasgupta BR. Effect of pH on the interaction of botulinum neurotoxins A, B and E with liposomes. Biochem J. 1989;259(1):47–53. 2719650 10.1042/bj2590047 PMC1138471

[11] Ruan Y, Yao L, Zhang B, Zhang S, Guo J. Nanoparticle-mediated delivery of neurotoxin-II to the brain with intranasal administration: an effective strategy to improve antinociceptive activity of neurotoxin. Drug Dev Ind Pharm. 2012;38(1):123–8. 21721852 10.3109/03639045.2011.592533

[12] Shrestha B. Nanotechnology for biosensor applications. Sustainable nanotechnology for environmental remediation: Elsevier; 2022:513-31.

[13] Girigoswami A, Ghosh MM, Pallavi P, Ramesh S, Girigoswami K. Nanotechnology in detection of food toxins–focus on the dairy products. Biointerface Res Appl Chem. 2021;11(6):14155–72.

[14] Nwankwegu AS, Li Y, Huang Y, Wei J, Norgbey E, Sarpong L, et al. Harmful algal blooms under changing climate and constantly increasing anthropogenic actions: the review of management implications. 3 Biotech. 2019;9:1–19. 10.1007/s13205-019-1976-1 PMC6856243 31832296

[15] Wang H, Zhu R, Zhang J, Ni L, Shen H, Xie P. A novel and convenient method for early warning of algal cell density by chlorophyll fluorescence parameters and its application in a highland lake. Front Plant Sci. 2018;9:869. 30002664 10.3389/fpls.2018.00869 PMC6031977

[16] Cabrera J, González PM, Puntarulo SÁ. Oxidative effects of the harmful algal blooms on primary organisms of the food web. 2019.

[17] Algal bloom and its economic impact: Publications Office; 2016.

[18] Young N, Sharpe RA, Barciela R, Nichols G, Davidson K, Berdalet E, et al. Marine harmful algal blooms and human health: A systematic scoping review. Harmful Algae. 2020;98:101901. 33129458 10.1016/j.hal.2020.101901

[19] Brenckman CM, Parameswarappa Jayalakshmamma M, Pennock WH, Ashraf F, Borgaonkar AD. A review of harmful algal blooms: causes, effects. Monitoring Prevention Methods. Water. 2025;17(13):1980.

[20] Nasso R, Pagliara V, D’Angelo S, Rullo R, Masullo M, Arcone R. Annurca apple polyphenol extract affects acetyl-cholinesterase and mono-amine oxidase in vitro enzyme activity. Pharmaceuticals. 2021;14(1):62. 33466604 10.3390/ph14010062 PMC7828649

[21] Ladant D, Marchot P, Diochot S, Prévost G, Popoff MR, Benoit E. Report from the 27th (Virtual) Meeting on Toxinology,“Toxins: Mr Hyde or Dr Jekyll?”, Organized by the French Society of Toxinology, 9–10 December 2021. MDPI; 2022. 10.3390/toxins14020110 PMC8876628 35202137

[22] Geffroy S, Lechat M-M, Le Gac M, Rovillon G-A, Marie D, Bigeard E, et al. From the sxtA4 gene to saxitoxin production: what controls the variability among Alexandrium minutum and Alexandrium pacificum strains? Front Microbiol. 2021;12:613199. 33717003 10.3389/fmicb.2021.613199 PMC7944994

[23] Popoff MR, Brüggemann H. Regulatory networks controlling neurotoxin synthesis in Clostridium botulinum and Clostridium tetani. Toxins. 2022;14(6):364. 35737025 10.3390/toxins14060364 PMC9229411

[24] Pinto A, Botelho MJ, Churro C, Asselman J, Pereira P, Pereira JL. A review on aquatic toxins-Do we really know it all regarding the environmental risk posed by phytoplankton neurotoxins? J Environ Manage. 2023;345:118769. 37597370 10.1016/j.jenvman.2023.118769

[25] Ayeni EA, Aldossary AM, Ayejoto DA, Gbadegesin LA, Alshehri AA, Alfassam HA, et al. Neurodegenerative diseases: Implications of environmental and climatic influences on neurotransmitters and neuronal hormones activities. Int J Environ Res Public Health. 2022;19(19):12495. 36231792 10.3390/ijerph191912495 PMC9564880

[26] Trainer VL, Moore SK, Hallegraeff G, Kudela RM, Clement A, Mardones JI, et al. Pelagic harmful algal blooms and climate change: Lessons from nature’s experiments with extremes. Harmful Algae. 2020;91:101591. 32057339 10.1016/j.hal.2019.03.009

[27] Turner AD, Lewis AM, Bradley K, Maskrey BH. Marine invertebrate interactions with harmful algal blooms–implications for one health. J Invertebr Pathol. 2021;186:107555. 33607127 10.1016/j.jip.2021.107555

[28] Brown AR, Lilley M, Shutler J, Lowe C, Artioli Y, Torres R, et al. Assessing risks and mitigating impacts of harmful algal blooms on mariculture and marine fisheries. Rev Aquac. 2020;12(3):1663–88.

[29] Lim CC, Yoon J, Reynolds K, Gerald LB, Ault AP, Heo S, et al. Harmful algal bloom aerosols and human health. EBioMedicine. 2023;93. 10.1016/j.ebiom.2023.104604 PMC10363441 37164781

[30] Yuan K-K, Li H-Y, Yang W-D. Marine algal toxins and public health: insights from shellfish and fish, the main biological vectors. Mar Drugs. 2024;22(11):510. 39590790 10.3390/md22110510 PMC11595774

[31] Gong Z, Huang Y, Hu X, Zhang J, Chen Q, Chen H. Recent progress in electrochemical nano-biosensors for detection of pesticides and mycotoxins in foods. Biosensors. 2023;13(1):140. 36671974 10.3390/bios13010140 PMC9856537

[32] Wilson M. Development of automated nucleic acid technologies for marine point of sample diagnostics: University of Southampton; 2021.

[33] Park JA, Seo Y, Sohn H, Park C, Min J, Lee T. Recent trends in biosensors based on electrochemical and optical techniques for cyanobacterial neurotoxin detection. BioChip J. 2022;16(2):146–57.

[34] Sujith S, Naresh R, Srivisanth BU, Sajeevan A, Rajaramon S, David H, et al. Aptamers: precision tools for diagnosing and treating infectious diseases. Front Cellular Infection Microbiol. 2024;14:1402932. 10.3389/fcimb.2024.1402932 PMC11461471 39386170

[35] Kim M, Eom H-J, Choi I, Hong J, Choi J. Graphene oxide-induced neurotoxicity on neurotransmitters, AFD neurons and locomotive behavior in Caenorhabditis elegans. Neurotoxicology. 2020;77:30–9. 31862286 10.1016/j.neuro.2019.12.011

[36] Tapeinos C. Graphene-based nanotechnology in neurodegenerative disorders. Adv NanoBiomed Res. 2021;1(3):2000059.

[37] Jodra A, López MÁ, Escarpa A. Disposable and reliable electrochemical magnetoimmunosensor for Fumonisins simplified determination in maize-based foodstuffs. Biosens Bioelectron. 2015;64:633–8. 10.1016/j.bios.2014.09.054 25441412

[38] Yu D, Zhang X, Qi Y, Ding S, Cao S, Zhu A, et al. Pb2+-modified graphene quantum dots as a fluorescent probe for biological aminothiols mediated by an inner filter effect. Sens Actuators B: Chem. 2016;235:394–400.

[39] Chopade SA, Anderson EL, Schmidt PW, Lodge TP, Hillmyer MA, Bühlmann P. Self-supporting, hydrophobic, ionic liquid-based reference electrodes prepared by polymerization-induced microphase separation. Acs Sens. 2017;2(10):1498–504. 28944667 10.1021/acssensors.7b00512

[40] Marei M, Kaht K, Roussel T, Keynton R, Baldwin R. Measurement of As (III) with in situ subtraction of background and interferent signals by double potential step-anodic stripping coulometry. Sens Actuators B: Chem. 2019;301:127005.

[41] Hassan RY. Advances in electrochemical nano-biosensors for biomedical and environmental applications: from current work to future perspectives. Sensors. 2022;22(19):7539. 36236638 10.3390/s22197539 PMC9573286

[42] Sumitha M, Xavier T. Recent advances in electrochemical biosensors–A brief review. Hybrid Adv. 2023;2:100023.

[43] Majdinasab M, Lamy de la Chapelle M, Marty JL. Recent progresses in optical biosensors for interleukin 6 detection. Biosensors. 2023;13(9):898. 37754132 10.3390/bios13090898 PMC10526799

[44] Jiao Y, Yang L, Hao J, Wen Y, Wang J, E H, et al. Rapid detection of saxitoxin using a nucleic acid aptamer biosensor based on graphene oxide as a fluorescence quencher. Toxins. 2025;17(9):430. 41003495 10.3390/toxins17090430 PMC12474413

[45] Zhao L, Guo H, Chen H, Zou B, Yang C, Zhang X, et al. A rapid and sensitive aptamer-based biosensor for amnesic shellfish toxin domoic acid. Bioengineering. 2022;9(11):684. 36421085 10.3390/bioengineering9110684 PMC9687209

[46] Kerry RG, Ukhurebor KE, Kumari S, Maurya GK, Patra S, Panigrahi B, et al. A comprehensive review on the applications of nano-biosensor-based approaches for non-communicable and communicable disease detection. Biomater Sci. 2021;9(10):3576–602. 34008586 10.1039/d0bm02164d

[47] Hu B, Ouyang SQ, Zhu YP, Lu XL, Ning Z, Jiao BH, et al. Brevetoxin aptamer selection and biolayer interferometry biosensor application. Toxins. 2024;16(10):411. 39453187 10.3390/toxins16100411 PMC11510897

[48] Sreejith S, Ajayan J, Radhika J, Reddy NU, Manikandan M. Recent advances in nano biosensors: An overview. Measurement. 2024:115073.

[49] Wei X, Wang S, Zhan Y, Kai T, Ding P. Sensitive identification of microcystin-LR via a reagent-free and reusable electrochemical biosensor using a methylene blue-labeled aptamer. Biosensors. 2022;12(8):556. 35892453 10.3390/bios12080556 PMC9332554

[50] Shreshtha R, Mulukutla A, Deb VK, Chauhan N, Jain U. Unveiling the cutting edge: recent developments in α-Synuclein nano-biosensor technology for precision diagnosis of Parkinson’s disease. BioNanoScience. 2025;15(1):171.

[51] Muhit R, Andrew W, Meredith S, Avni AA. Rapid and low-cost field toxin analysis to monitor harmful algal blooms. Biosens Nanotheranostics. 2025;4(1):1.

[52] Rather SA, Mustafa RA, Ashraf MV, Hannan Khan M, Ahmad S, Wani ZA. Implications of nano-biosensors in the early detection of neuroparasitic diseases. theranostic applications of nanotechnology in neurological disorders: Springer; 2024:43-83.

[53] Samal S, Mohanty RP, Mohanty PS, Giri MK, Pati S, Das B. Implications of biosensors and nanobiosensors for the eco-friendly detection of public health and agro-based insecticides: A comprehensive review. Heliyon. 2023. 10.1016/j.heliyon.2023.e15848. 37206035 10.1016/j.heliyon.2023.e15848 PMC10189192

[54] Jaric S, Bajaj A, Vukic V, Gadjanski I, Abdulhalim I, Bobrinetskiy I. Label-free direct detection of cylindrospermopsin via graphene-enhanced surface plasmon resonance aptasensor. Toxins. 2023;15(5):326. 37235360 10.3390/toxins15050326 PMC10220545

[55] Tang X, Jiang H, Wen R, Xue D, Zeng W, Han Y, et al. Advancements and challenges on SERS-based multimodal biosensors for biotoxin detection. Trends Food Sci Technol. 2024. 10.1016/j.tifs.2024.104672.

[56] Mills C, Dillon MJ, Kulabhusan PK, Senovilla-Herrero D, Campbell K. Multiplex lateral flow assay and the sample preparation method for the simultaneous detection of three marine toxins. Environ Sci Technol. 2022;56(17):12210–7. 35951987 10.1021/acs.est.2c02339 PMC9454242

[57] Zhang W, Dixon MB, Saint C, Teng KS, Furumai H. Electrochemical biosensing of algal toxins in water: the current state-of-the-art. ACS Sensors. 2018;3(7):1233–45. 29974739 10.1021/acssensors.8b00359

[58] Kumar H, Yadav DK. Relevance of nano biosensors in eco hazards. Environ Wellness.105.

[59] Bilge S, Bakirhan NK. Green applications of electrochemical biosensors. In: Manjunatha JG, editor. Biosensing technology for human health: eco-friendly materials and real-world applications, vol. 27. Royal Society of Chemistry; 2024. p. 0.

[60] Zahir M, Su Y, Shahzad MI, Ayub G, Rehman SU, Ijaz J. A review on monitoring, forecasting, and early warning of harmful algal bloom. Aquaculture. 2024;593:741351.

[61] Kishore A, Singh C, Kaur G. Bio‐nanomaterials: An introduction. bio‐nanomaterials in environmental remediation: industrial applications. 2025:1-46.

[62] Aguilar-Pérez K, Heya M, Parra-Saldívar R, Iqbal HM. Nano-biomaterials in-focus as sensing/detection cues for environmental pollutants. Case Studies Chem Environ Eng. 2020;2:100055.

[63] Menaa F, Wijesinghe U, Thiripuranathar G, Althobaiti NA, Albalawi AE, Khan BA, et al. Marine algae-derived bioactive compounds: a new wave of nanodrugs. Mar Drugs. 2021;19(9):484. 34564146 10.3390/md19090484 PMC8469996

[64] Bedi N, Srivastava DK, Srivastava A, Mahapatra S, Dkhar DS, Chandra P, et al. Marine biological macromolecules as matrix material for biosensor fabrication. Biotechnol Bioeng. 2022;119(8):2046–63. 35470439 10.1002/bit.28122

[65] Oliveira C, Neves NM, Reis RL, Martins A, Silva TH. A review on fucoidan antitumor strategies: From a biological active agent to a structural component of fucoidan-based systems. Carbohyd Polym. 2020;239:116131. 10.1016/j.carbpol.2020.116131 32414455

[66] Hu M, Tang Y, He X, Liu K, Qin L, Wang X, et al. Enzyme-integrated hydrogels for advanced biological applications. 2025. 10.1021/polymscitech.5c00076 PMC13052696 42253785

[67] Venkatesan J, Murugan SS, Seong GH. Fucoidan-based nanoparticles: Preparations and applications. Int J Biol Macromol. 2022;217:652–67. 35841962 10.1016/j.ijbiomac.2022.07.068

[68] Qamar SA, Junaid M, Riasat A, Jahangeer M, Bilal M, Mu BZ. Carrageenan-based hybrids with biopolymers and nano-structured materials for biomimetic applications. Starch-Stärke. 2024;76(1–2):2200018.

[69] Cotruvo JA. WHO issues updated draft microcystin algal toxin guidance. J AWWA. 2020;112(8):84–6.

[70] Moreira JB, Santos TD, Cruz CG, Silveira JT, Carvalho LF, Morais MG, et al. Algal polysaccharides-based nanomaterials: general aspects and potential applications in food and biomedical fields. Polysaccharides. 2023;4(4):371–89.

[71] O’Connor J, Garcia-Vaquero M, Meaney S, Tiwari BK. Bioactive peptides from algae: traditional and novel generation strategies, structure-function relationships, and bioinformatics as predictive tools for bioactivity. Mar Drugs. 2022;20(5):317. 35621968 10.3390/md20050317 PMC9145204

[72] Chollet L, Saboural P, Chauvierre C, Villemin J-N, Letourneur D, Chaubet F. Fucoidans in nanomedicine. Mar Drugs. 2016;14(8):145. 27483292 10.3390/md14080145 PMC4999906

[73] Hassan RA, Heng LY, Tan LL. Novel DNA Biosensor for Direct Determination of Carrageenan. Sci Rep [Internet]. 2019 2019/04//; 9(1):[6379 p.]. Available from: 10.1038/s41598-019-42757-y PMC6478878 31015498

[74] Liu X, Zhang Q, Knoll W, Liedberg B, Wang Y. Rational design of functional peptide–gold hybrid nanomaterials for molecular interactions. Adv Mater. 2020;32(37):2000866. 10.1002/adma.202000866 32743897

[75] Wang P, Zhang Y, Hu J, Tan BK. Bioactive peptides from marine organisms. Protein Pept Lett. 2024;31(8):569–85. 39253911 10.2174/0109298665329840240816062134

[76] Kim SM, Xu P, Hyun MS, Park JP, Park CY, Park TJ. Development of an electrochemical biosensor for tetrodotoxin using specific binding peptide on Polypyrrole/Au nanoparticle-modified electrodes. BioChip J. 2024;18(3):495–504.

[77] Bilibana MP, Citartan M, Fuku X, Jijana AN, Mathumba P, Iwuoha E. Aptamers functionalized hybrid nanomaterials for algal toxins detection and decontamination in aquatic system: current progress, opportunities, and challenges. Ecotoxicol Environ Saf. 2022;232:113249. 35104779 10.1016/j.ecoenv.2022.113249

[78] Paul AA, Marks RS, Markus V, Kristollari K. Alginate-based applications in biotechnology with a special mention to biosensors. In: Aguiar Severo I, Bellin Mariano A, Vargas JVC, editors. Alginate-applications and future perspectives. London: IntechOpen; 2023.

[79] Ismail GA, El-Sheekh MM, Samy RM, Gheda SF. Antimicrobial, antioxidant, and antiviral activities of biosynthesized silver nanoparticles by phycobiliprotein crude extract of the cyanobacteria Spirulina platensis and Nostoc linckia. Bionanoscience. 2021;11:355–70.

[80] Wu X-J, Dong D-W, Qu J-Y, Han Y, You Z-Y, Li P-P, et al. An enzyme-fused phycobiliprotein synthesis system developed for visual whole-cell biosensors for the detection of cadmium during wastewater treatment. Environ Technol Innov. 2024;36:103882.

[81] Chen H, Deng J, Li L, Liu Z, Sun S, Xiong P. Recent progress of natural and recombinant phycobiliproteins as fluorescent probes. Mar Drugs. 2023;21(11):572. 37999396 10.3390/md21110572 PMC10672124

[82] Makshakova ON, Bogdanova LR, Faizullin DA, Ermakova EA, Zuev YF. Sulfated polysaccharides as a fighter with protein non-physiological aggregation: the role of polysaccharide flexibility and charge density. Int J Mol Sci. 2023;24(22):16223. 38003413 10.3390/ijms242216223 PMC10671430

[83] Veraldi N, Quadri ID, Van De Looij Y, Modernell LM, Sinquin C, Zykwinska A, et al. Low-molecular weight sulfated marine polysaccharides: Promising molecules to prevent neurodegeneration in mucopolysaccharidosis IIIA? Carbohyd Polym. 2023;320:121214. 10.1016/j.carbpol.2023.121214 37659814

[84] Nagahawatta DP, Liyanage NM, Jayawardena TU, Yang F, Jayawardena HHACK, Kurera MJMS, et al. Functions and values of sulfated polysaccharides from seaweed. Algae. 2023;38(4):217–40.

[85] Kabir MT, Rahman MH, Shah M, Jamiruddin MR, Basak D, Al-Harrasi A, et al. Therapeutic promise of carotenoids as antioxidants and anti-inflammatory agents in neurodegenerative disorders. Biomed Pharmacother. 2022;146:112610. 35062074 10.1016/j.biopha.2021.112610

[86] Lakey-Beitia J, Kumar DJ, Hegde ML, Rao K. Carotenoids as novel therapeutic molecules against neurodegenerative disorders: Chemistry and molecular docking analysis. Int J Mol Sci. 2019;20(22):5553. 31703296 10.3390/ijms20225553 PMC6888440

[87] Tam CC, Flannery AR, Cheng LW. A rapid sensitive, and portable biosensor assay for the detection of botulinum neurotoxin serotype a in complex food matrices. Toxins. 2018;10(11):476. 30445734 10.3390/toxins10110476 PMC6266793

[88] McPartlin DA, Lochhead MJ, Connell LB, Doucette GJ, O’Kennedy RJ. Use of biosensors for the detection of marine toxins. Essays Biochem. 2016;60(1):49–58. 27365035 10.1042/EBC20150006 PMC4986468

[89] Song J, Xu Z, Chen Y, Guo J. Nanoparticles, an emerging control method for harmful algal blooms: current technologies, challenges, and perspectives. Nanomaterials. 2023;13(16):2384. 37630969 10.3390/nano13162384 PMC10457966

[90] Wu D, Li R, Zhang F, Liu J. A review on drone-based harmful algae blooms monitoring. Environ Monit Assess. 2019;191:1–11. 10.1007/s10661-019-7365-8 30852736

[91] Tseytlin IN, Antrim AK, Gong P. Nanoparticles for mitigation of harmful cyanobacterial blooms. Toxins. 2024;16(1):41. 38251256 10.3390/toxins16010041 PMC10819728

[92] Alprol AE, Mansour AT, Abdelwahab AM, Ashour M. Advances in green synthesis of metal oxide nanoparticles by marine algae for wastewater treatment by adsorption and photocatalysis techniques. Catalysts. 2023;13(5):888.

[93] Ahmad MA, Adeel M, Shakoor N, Javed R, Ishfaq M, Peng Y, et al. Modifying engineered nanomaterials to produce next generation agents for environmental remediation. Sci Total Environ. 2023;894:164861. 37343875 10.1016/j.scitotenv.2023.164861

[94] Gudainiyan J, Kumar R, Singh D, Sing DP, Shrivastava A, Srivastava AP, et al., editors. Nanotechnology-enabled Solutions for Water Purification and Environmental Sustainability. E3S Web of Conferences; 2024: EDP Sciences.

[95] Awlqadr FH, Altemimi AB, Qadir SA, Salih TAH, Alkanan ZT, AlKaisy QH, et al. Emerging trends in nano-sensors: A new frontier in food safety and quality assurance. 2025;11(1). 10.1016/j.heliyon.2024.e41181 PMC11728908 39807502

[96] Zhao Y, Song J, Yang Q, Li Y, Liu Z, Yang F. Real-time visualization of carbon quantum dot transport in homogeneous and heterogeneous porous media. Environ Sci Nano. 2024;11(12):4743–53.

[97] Liu S, Xiao J, Ma L, Li X. Study of the biological glutathione in algae by established quantum dot covalent coupling system. J Environ Eng. 2023;149(7):04023031.

[98] Li C, Zhu Z, Yao J, Chen Z, Huang Y. Perspectives in Aptasensor-based portable detection for Biotoxins. Molecules. 2024;29(20):4891. 39459259 10.3390/molecules29204891 PMC11510259

[99] Zhao L, Guo H, Chen H, Zou B, Yang C, Zhang X, et al. A rapid and sensitive aptamer-based biosensor for amnesic shellfish toxin domoic acid. Bioengineering (Basel, Switzerland). 2022;9(11). 10.3390/bioengineering9110684 PMC9687209 36421085

[100] Vogiazi V, De La Cruz A, Mishra S, Shanov V, Heineman WR, Dionysiou DD. A comprehensive review: Development of electrochemical biosensors for detection of cyanotoxins in freshwater. ACS sensors. 2019;4(5):1151–73. 31056912 10.1021/acssensors.9b00376 PMC6625642

[101] Bano K, Khan WS, Cao C, Khan RF, Webster TJ. Biosensors for detection of marine toxins. Nanobiosensors: From Design to Applications. 2020:329–56.

[102] Stevenson LM, Adeleye AS, Su Y, Zhang Y, Keller AA, Nisbet RM. Remediation of cadmium toxicity by sulfidized nano-iron: the importance of organic material. ACS Nano. 2017;11(10):10558–67. 28985677 10.1021/acsnano.7b05970

[103] Hlongwane GN, Sekoai PT, Meyyappan M, Moothi K. Simultaneous removal of pollutants from water using nanoparticles: A shift from single pollutant control to multiple pollutant control. Sci Total Environ. 2019;656:808–33. 30530150 10.1016/j.scitotenv.2018.11.257

[104] Wang B, Wu D, Chu KH, Ye L, Yip HY, Cai Z, et al. Removal of harmful alga, Chattonella marina, by recyclable natural magnetic sphalerite. J Hazard Mater. 2017;324:498–506. 27847251 10.1016/j.jhazmat.2016.11.018

[105] Sujatha G, Padmavathi S, Padmaja V, Pushpa Latha A. Plant growth promoting cyanobacteria—based nanoparticles in agriculture. nanofertilizers in agriculture: synthesis, mechanisms, and effect on plants: Springer; 2025. p. 141–61.

[106] Kwon Y, Yoon Y, Jang M, Kang S, Park C, Lee T. Recent advances in cyanobacterial cytotoxin biosensors focused on cylindrospermopsin. Chemosensors. 2023;11(7):401.

[107] Valenzuela-Amaro HM, Aguayo-Acosta A, Meléndez-Sánchez ER, de la Rosa O, Vázquez-Ortega PG, Oyervides-Muñoz MA, et al. Emerging applications of nanobiosensors in pathogen detection in water and food. Biosensors. 2023;13(10):922. 37887115 10.3390/bios13100922 PMC10605657

[108] Onamade AO, Asaju OA, Adewumi BJ. Challenges and future prospects of smart nanomaterials for environmental remediation. Smart Nanomaterials for Environmental Applications. 2025:757–80.

[109] Honeychurch KC, Piano M. Sensors for environmental monitoring and food safety. MDPI; 2022. p. 366. 10.3390/bios12060366 PMC9220911 35735513

[110] Nachiappan K, Velmurugan K, Gurumurthy Vedavalli H, Krishnan A, Sekar G, Balakrishnan Y, et al. An integrated approach of green nanotechnology in wastewater remediation using algae. Materials Technology for the Energy and Environmental Nexus, Volume 2: IOP Publishing Bristol, UK; 2023. p. 12–1--8.

[111] Chen F, Xiao Z, Yue L, Wang J, Feng Y, Zhu X, et al. Algae response to engineered nanoparticles: current understanding, mechanisms and implications. Environ Sci Nano. 2019;6(4):1026–42.

[112] Rodríguez-González V, Alfaro SO, Torres-Martínez L, Cho S-H, Lee S-W. Silver–TiO2 nanocomposites: synthesis and harmful algae bloom UV-photoelimination. Appl Catal B. 2010;98(3–4):229–34.

[113] Von Tress N. Effectiveness of titanium and iron nanoparticles in treating m. aeruginosa for harmful algal bloom remediation. 2019.

[114] Kokkinos P, Mantzavinos D, Venieri D. Current trends in the application of nanomaterials for the removal of emerging micropollutants and pathogens from water. Molecules. 2020;25(9):2016. 32357416 10.3390/molecules25092016 PMC7248945

[115] Fan G, Chen Z, Hong L, Du B, Yan Z, Zhan J, et al. Simultaneous removal of harmful algal cells and toxins by a Ag2CO3-N: GO photocatalyst coating under visible light. Sci Total Environ. 2020;741:140341. 32615428 10.1016/j.scitotenv.2020.140341

[116] Huang T, Sui M, Yan X, Zhang X, Yuan Z. Anti-algae efficacy of silver nanoparticles to Microcystis aeruginosa: Influence of NOM, divalent cations, and pH. Colloids Surf, A. 2016;509:492–503.

[117] Kordahi MA, Ayoub GM, Zayyat RM. A critical review of current research on cyanobacterial cells and associated toxins in aquatic environments: occurrence, impact, and treatment methods. J Environ Chem Eng. 2024. 10.1016/j.jece.2024.113931.

[118] Corsi I, Bellingeri A, Eliso MC, Grassi G, Liberatori G, Murano C, et al. Eco-interactions of engineered nanomaterials in the marine environment: towards an eco-design framework. Nanomaterials. 2021;11(8):1903. 34443734 10.3390/nano11081903 PMC8398366

[119] Deidda I, Russo R, Bonaventura R, Costa C, Zito F, Lampiasi N. Neurotoxicity in marine invertebrates: An update. Biology. 2021;10(2):161. 33670451 10.3390/biology10020161 PMC7922589

[120] Bownik A, Wlodkowic D. Applications of advanced neuro-behavioral analysis strategies in aquatic ecotoxicology. Sci Total Environ. 2021;772:145577. 33770877 10.1016/j.scitotenv.2021.145577

[121] Botelho MJ, Milinovic J, Bandarra NM, Vale C. Alzheimer’s disease and toxins produced by marine dinoflagellates: An issue to explore. Mar Drugs. 2022;20(4):253. 35447926 10.3390/md20040253 PMC9029327

[122] Katikou P, Gokbulut C, Kosker AR, Campàs M, Ozogul F. An updated review of tetrodotoxin and its peculiarities. Mar Drugs. 2022;20(1):47. 35049902 10.3390/md20010047 PMC8780202

[123] Venkatesh B, Lu SQ, Dandona N, See SL, Brenner S, Soong TW. Genetic basis of tetrodotoxin resistance in pufferfishes. Current Biol. 2005;15(22):2069–72. 10.1016/j.cub.2005.10.068 16303569

[124] Guillotin S, Delcourt N. Marine neurotoxins’ effects on environmental and human health: an OMICS overview. Mar Drugs. 2021;20(1):18. 35049872 10.3390/md20010018 PMC8778346

[125] Mackieh R, Abou-Nader R, Wehbe R, Mattei C, Legros C, Fajloun Z, et al. Voltage-gated sodium channels: a prominent target of marine toxins. Mar Drugs. 2021;19(10):562. 34677461 10.3390/md19100562 PMC8537899

[126] Turcio R, Di Matteo F, Capolupo I, Ciaglia T, Musella S, Di Chio C, et al. Voltage-gated K+ channel modulation by marine toxins: pharmacological innovations and therapeutic opportunities. Mar Drugs. 2024;22(8):350. 39195466 10.3390/md22080350 PMC11355921

[127] Chen R, Liu Y, Qian L, Yi M, Yin H, Wang S, et al. Sodium channels as a new target for pain treatment. Front Pharmacol. 2025;16:1573254. 40206072 10.3389/fphar.2025.1573254 PMC11979154

[128] Pope E, Bigelow L, Bernard P. Glutamatergic neurotransmission and toxicity: domoic acid and kainic acid (Glutamic Acid Analogs). 2024.

[129] Jiang R, Fan Z, Li X, Yang J, Sun M, Jiao B, et al. Molecular and cellular mechanisms underlying domoic acid-induced neurotoxicity and therapeutic drugs: a comprehensive review. Int J Mol Sci. 2026;27(2):867. 41596513 10.3390/ijms27020867 PMC12841449

[130] Petroff RL, Williams C, Li J-L, MacDonald JW, Bammler TK, Richards T, et al. Prolonged, low-level exposure to the marine toxin, domoic acid, and measures of neurotoxicity in nonhuman primates. Environ Health Perspect. 2022;130(9):097003. 36102641 10.1289/EHP10923 PMC9472675

[131] Pérez-Gomez A, Tasker RA. Domoic acid as a neurotoxin. Handbook of neurotoxicity: Springer; 2023. p. 873–97.

[132] Turcatel GA, Moura SJAo. Glutamate receptor agonists as triggers of neurotoxicity: decoding pathways of five neurotoxins and potential therapeutic targets. 2025. 10.1021/acsomega.5c05841 PMC12809595 41552526

[133] Sharma S, Rana P, Dhingra N, Kaur T. Insights into ion channels to identify drug target for neuropathic pain management. Inflammopharmacol. 2025;33(8):4455–75. 10.1007/s10787-025-01899-4 40794371

[134] Mishra S, Mishra Y, Kumar A. Marine-derived bioactive compounds for neuropathic pain: pharmacology and therapeutic potential. Naunyn Schmiedebergs Arch Pharmacol. 2025. 10.1007/s00210-024-03667-7. 39797987 10.1007/s00210-024-03667-7

[135] Khotimchenko YS, Silachev DN, Katanaev VL. Marine natural products from the Russian Pacific as sources of drugs for neurodegenerative diseases. Mar Drugs. 2022;20(11):708. 36421986 10.3390/md20110708 PMC9697637

[136] Jia C, Chai J, Zhang S, Sun Y, He L, Sang Z, et al. The Advancements of marine natural products in the treatment of alzheimer’s disease: a study based on cell and animal experiments. Mar Drugs. 2025;23(3):91. 40137277 10.3390/md23030091 PMC11943648

[137] Wendimu MY. Anti-Inflammatory and neuroprotective mechanisms of regulator of G protein signaling 10 (RGS10) in Microglia: University of Georgia; 2022.

[138] Mohamed HM. Sensors and biosensors for environment contaminants. Nanosensor technologies for environmental monitoring: Springer; 2020. p. 109–34.

[139] Timilsina SS, Jolly P, Durr N, Yafia M, Ingber DE. Enabling multiplexed electrochemical detection of biomarkers with high sensitivity in complex biological samples. Accounts Chem Res. 2021;54(18):3529–39. 10.1021/acs.accounts.1c00382 34478255

[140] Zhu X, Zhao Y, Wu L, Gao X, Huang H, Han Y, et al. Advances in biosensors for the rapid detection of marine biotoxins: current status and future perspectives. Biosensors. 2024;14(4):203. 38667196 10.3390/bios14040203 PMC11048312

[141] Serrano PC, Nunes GE, Avila LB, Reis CPS, Gomes AMC, Reis FT, et al. Electrochemical impedance biosensor for detection of saxitoxin in aqueous solution. Anal Bioanal Chem. 2021;413(25):6393–9. 34389880 10.1007/s00216-021-03603-1

[142] Li J, Xu Z, Zhang Z, Liu R, Zhu Y, Lu X, et al. Aptamer-based dual-cascade signal amplification system lights up G-Quadruplex dimers for ultrasensitive detection of domoic acid. Mar Drugs. 2026;24(1):50. 41590747 10.3390/md24010050 PMC12843330

[143] Liu Z, Huo Y, Hu Z, Xu Y, Hao B, Yao X, et al. Nanomaterials-enabled biosensing platforms for microcystin-LR detection: a review of analytical advancements. Anal Bioanal Chem. 2025;417(24):5363–88. 40542893 10.1007/s00216-025-05968-z

[144] Cui Z, Jiang F, Li L, Chi Z, Liu C. Advances in biomedical applications of hydrogels from seaweed-derived sulfated polysaccharides: Carrageenan, Fucoidan, and Ulvan. J Ocean University of China. 2024;23(5):1329–46.

[145] Silveri F, Della Pelle F, Merola C, Scroccarello A, Trabucco F, Amorena M, et al. Algae-paper integrated sensor for bisphenol determination in zebrafish embryos. Sci Total Environ. 2025;1002:180529. 41005175 10.1016/j.scitotenv.2025.180529

[146] Gao X, Wang H, Chen K, Guo Y, Zhou J, Xie W. Toxicological and pharmacological activities, and potential medical applications, of marine algal toxins. Int J Mol Sci. 2024;25(17):9194. 39273145 10.3390/ijms25179194 PMC11394994

[147] Silva J, Alves C, Soledade F, Martins A, Pinteus S, Gaspar H, et al. Marine-derived components: can they be a potential therapeutic approach to Parkinson’s disease? Mar Drugs. 2023;21(8):451. 37623732 10.3390/md21080451 PMC10455662

[148] Lee H, Lytton-Jean AK, Chen Y, Love KT, Park AI, Karagiannis ED, et al. Molecularly self-assembled nucleic acid nanoparticles for targeted in vivo siRNA delivery. Nat Nanotechnol. 2012;7(6):389–93. 22659608 10.1038/nnano.2012.73 PMC3898745

[149] Chałupniak A, Morales-Narváez E, Merkoçi A. Micro and nanomotors in diagnostics. Adv Drug Delivery Rev. 2015;95:104–16. 10.1016/j.addr.2015.09.004 26408790

[150] Saraiva C, Praça C, Ferreira R, Santos T, Ferreira L, Bernardino LJ. Nanoparticle-mediated brain drug delivery: Overcoming blood–brain barrier to treat neurodegenerative diseases. J Controlled Release. 2016;235:34–47. 10.1016/j.jconrel.2016.05.044 27208862

[151] Pinheiro RG, Coutinho AJ, Pinheiro M, Neves AR. Nanoparticles for targeted brain drug delivery: what do we know? Int J Mol Sci. 2021;22(21):11654. 34769082 10.3390/ijms222111654 PMC8584083

[152] Kai M, Shen W-T, Yu Y, Wang D, Zhang JA, Wang S, et al. Dual-modal cellular nanoparticles for continuous neurotoxin detoxification. Nano Lett. 2024;24(24):7515–23. 10.1021/acs.nanolett.4c01798 38855905

[153] Wang S, Wang D, Shen WT, Kai M, Yu Y, Peng Y, et al. Protein-loaded cellular nanosponges for dual-biomimicry neurotoxin countermeasure. Small. 2024;20(14):2309635. 10.1002/smll.202309635 37990378

[154] Louzao MC, Vilariño N, Vale C, Costas C, Cao A, Raposo-Garcia S, et al. Current trends and new challenges in marine phycotoxins. Mar Drugs. 2022;20(3):198. 35323497 10.3390/md20030198 PMC8950113

[155] Flores-Holguín N, Salas-Leiva JS, Núñez-Vázquez EJ, Tovar-Ramírez D, Glossman-Mitnik D. Marine toxins as pharmaceutical treasure troves: a focus on saxitoxin derivatives from a computational point of view. Molecules. 2024;29(1):275. 38202857 10.3390/molecules29010275 PMC10780485

[156] Bernatoniene J, Plieskis M, Petrikonis K. Pharmaceutical 3D printing technology integrating nanomaterials and nanodevices for precision neurological therapies. Pharmaceutics. 2025;17(3):352. 40143015 10.3390/pharmaceutics17030352 PMC11945809

[157] Sabir F, Ain QU, Rahdar A, Yang Z, Barani M, Bilal M, et al. Functionalized nanoparticles in drug delivery: Strategies to enhance direct nose-to-brain drug delivery via integrated nerve pathways. Synthesis Appl Nanoparticles. 2022:455–85.

[158] Nevins S, McLoughlin CD, Oliveros A, Stein JB, Rashid MA, Hou Y, et al. Nanotechnology approaches for prevention and treatment of chemotherapy-induced neurotoxicity, neuropathy, and cardiomyopathy in breast and ovarian cancer survivors. Small. 2024;20(41):2300744. 10.1002/smll.202300744 PMC10576016 37058079

[159] Teixeira LM, Reis CP, Pacheco R. Marine-derived compounds combined with nanoparticles: a focus on the biomedical and pharmaceutical sector. Mar Drugs. 2025;23(5):207. 40422797 10.3390/md23050207 PMC12113654

[160] Singh SK, Sharma A, Sharma L, Sundaram S. Green synthesis of algal nanoparticles: harnessing nature’s biofactories for sustainable nanomaterials. Biogenic wastes-enabled nanomaterial synthesis: applications in environmental sustainability: Springer; 2024. p. 257-84.

[161] Kalyanam M, Sunder KS, Mishra B, Das M, Dey H, Govindugari VL. Algal-mediated nanoparticle synthesis for environmental sustainability. Nano-microbiology for Sustainable Development: Springer; 2025. p. 53–69.

[162] Ahmed I, Mirza AU, Banday JA, Mir FA, Zainab K, Ahmed S. Marine-based nanoparticles for bioimaging. Marine Biopolymers: Elsevier; 2025. p. 385–421.

[163] Siddiqi KS, ur Rahman A, Tajuddin, Husen A. 2016 Biogenic fabrication of iron/iron oxide nanoparticles and their application. Nanoscale Res lett. 11(1):498 10.1186/s11671-016-1714-0 PMC5106417 27837567

[164] Periyathambi P, Vedakumari WS, Bojja S, Kumar SB, Sastry TP. Green biosynthesis and characterization of fibrin functionalized iron oxide nanoparticles with MRI sensitivity and increased cellular internalization. Mater Chem Phys. 2014;148(3):1212–20.

[165] Anti-Inflammatory A-OJPCR, Challenges, prospects. Biomedical applications of algae-synthesized nanoparticles: Drug. 2025:99.

[166] Abdelmonem M, Albert EL, Alhadad MA, Abdullah CAJPN. Plant-polyphenol-mediated synthesis of magnetic biocompatible iron oxide nanoparticles for diagnostic imaging and management of neurodegenerative diseases. Precision Nanomed. 2024;7(1).

[167] Palaniappan P, Surendirakumar K, Ravi M, Ramesh R. Exploring the effects of seaweed synthesized nanoparticles on human cancer cell lines. cytotoxicity-a crucial toxicity test for in vitro experiments: IntechOpen; 2025.

[168] Kumarasinghe H, Virajini MT, Gunathilaka M. Biomedical advances in high-throughput screening of marine-derived drugs in cancer therapy. Transformative applied research in computing, engineering, science and technology: CRC Press; 2025:36-45.

[169] Atmaca H, Oguz F, Ilhan S. Drug delivery systems for cancer treatment: a review of marine-derived polysaccharides. Curr Pharm Des. 2022;28(13):1031–45. 35152862 10.2174/1381612828666220211153931

[170] Saranyadevi S, Gangadhar L, Sana SS, Kadem AA. Synthesis, characterization, and application of nanoparticles from medicinal plant-based carotenoids. Secondary Metabolites from Medicinal Plants: CRC Press; 2023. p. 151–76.

[171] Jafari M, Sriram V, Xu Z, Harris GM, Lee JY. Fucoidan-doxorubicin nanoparticles targeting P-selectin for effective breast cancer therapy. Carbohyd Polym. 2020;249:116837. 10.1016/j.carbpol.2020.116837 32933681

[172] Shu G, Lu C, Wang Z, Du Y, Xu X, Xu M, et al. Fucoidan-based micelles as P-selectin targeted carriers for synergistic treatment of acute kidney injury. Nanomed: Nanotechnol, Biol Med. 2021;32:102342. 10.1016/j.nano.2020.102342 33253922

[173] Silva M, Avni D, Varela J, Barreira L. The Ocean’s pharmacy: Health discoveries in marine algae. Molecules. 2024;29(8):1900. 38675719 10.3390/molecules29081900 PMC11055030

[174] Ersoydan S, Rustemeyer T. Investigating the anti-inflammatory activity of various brown algae species. Mar Drugs. 2024;22(10):457. 39452865 10.3390/md22100457 PMC11509244

[175] Berri M, Olivier M, Holbert S, Dupont J, Demais H, Le Goff M, et al. Ulvan from Ulva armoricana (Chlorophyta) activates the PI3K/Akt signalling pathway via TLR4 to induce intestinal cytokine production. Algal Res. 2017;28:39–47.

[176] Hu C-MJ, Zhang LJBp. Nanoparticle-based combination therapy toward overcoming drug resistance in cancer. Biochem Pharmacol. 2012;83(8):1104–11. 22285912 10.1016/j.bcp.2012.01.008

[177] Chaudhary R, Nawaz K, Khan AK, Hano C, Abbasi BH, Anjum S. An overview of the algae-mediated biosynthesis of nanoparticles and their biomedical applications. Biomolecules. 2020;10(11):1498. 33143289 10.3390/biom10111498 PMC7693774

[178] Premarathna K, Lau SY, Chiong T, Show P-L, Vithanage M, Lam MKJCT, et al. Greening up the fight against emerging contaminants: algae-based nanoparticles for water remediation. Clean Technologies Environ Policy. 2025;27(10):5825–42.

[179] Sethi S, Jonwal H. Harnessing algae and nanotechnology for efficient remediation of environmental pollutants. Phyconanotechnology: current research, challenges, and prospects: Springer; 2025. p. 129-44.

[180] James J, Verma M, Sharma N. Nanotechnology-driven improvisation of red algae-derived carrageenan for industrial and bio-medical applications. World J Microbiol Biotechnol. 2024;40(1):4. 10.1007/s11274-023-03787-x 37923917

[181] Algar WR, Massey M, Rees K, Higgins R, Krause KD, Darwish GH, et al. Photoluminescent nanoparticles for chemical and biological analysis and imaging. Chem Rev. 2021;121(15):9243–358. 34282906 10.1021/acs.chemrev.0c01176

[182] Casanova Y, Negro S, Barcia E. Application of neurotoxin-and pesticide-induced animal models of Parkinson’s disease in the evaluation of new drug delivery systems. Acta Pharm. 2022;72(1):35–58. 36651528 10.2478/acph-2022-0008

[183] Mishra A. Neurotoxins from marine dinoflagellates and diatoms: source, structure and toxic effects. Bio vet innovator magazine. 2025;2.

[184] Drori E, Patel D, Coopersmith S, Rahamim V, Drori C, Jadhav SS, et al. Algae-based nanoparticles for oral drug delivery systems. Mar Drugs. 2024;22(3):98. 38535439 10.3390/md22030098 PMC10971847

[185] Feng S, Xie X, Liu J, Li A, Wang Q, Guo D, et al. A potential paradigm in CRISPR/Cas systems delivery: at the crossroad of microalgal gene editing and algal-mediated nanoparticles. J Nanobiotechnol. 2023;21(1):370. 10.1186/s12951-023-02139-z PMC10563294 37817254

[186] Khan F, Shahid A, Zhu H, Wang N, Javed MR, Ahmad N, et al. Prospects of algae-based green synthesis of nanoparticles for environmental applications. Chemosphere. 2022;293:133571. 35026203 10.1016/j.chemosphere.2022.133571

[187] Panlilio JM, Aluru N, Hahn ME. Developmental neurotoxicity of the harmful algal bloom toxin domoic acid: cellular and molecular mechanisms underlying altered behavior in the zebrafish model. Environ Health Perspect. 2020;128(11):117002. 33147070 10.1289/EHP6652 PMC7641300

[188] Wang D-Z. Neurotoxins from marine dinoflagellates: a brief review. Mar Drugs. 2008;6(2):349–71. 18728731 10.3390/md20080016 PMC2525493

[189] Raulo S, Samanta A, Baliarsingh SK, Sarma VVSS, Joseph S, Nair TMB, et al. Determining chlorophyll-a thresholds for characterizing algal bloom conditions: An ocean colour remote sensing approach. Sci Total Environ. 2025;961:178353. 39793139 10.1016/j.scitotenv.2024.178353

[190] Khan RM, Salehi B, Mahdianpari M, Mohammadimanesh F, Mountrakis G, Quackenbush LJ. A meta-analysis on harmful algal bloom (HAB) detection and monitoring: a remote sensing perspective. Remote Sens. 2021;13(21):4347.

[191] Carias J, Vásquez-Lavín F, Barrientos M, Ponce Oliva RD, Gelcich S. Economic valuation of harmful algal blooms (HAB): methodological challenges, policy implications, and an empirical application. J Environ Manage. 2024;365:121566. 38909578 10.1016/j.jenvman.2024.121566

[192] Kouakou CRC, Poder TG. Economic impact of harmful algal blooms on human health: a systematic review. J Water Health. 2019;17(4):499–516. 31313990 10.2166/wh.2019.064

[193] Shen H, Cui Y, Liang S, Zhou S, Li Y, Wu Y, et al. A high-throughput biosensing approach for rapid screening of compounds targeting the hNav11 channel: marine toxins as a case study. Mar Drugs. 2025;23(3):119. 40137305 10.3390/md23030119 PMC11943507

[194] Srinivasan S, Letran K, Nasir S, Sung K, Lam J, Wang Y, et al. Microfluidic nanobiosensor detection of botulinum neurotoxin serotype A. ACS Omega. 2025;10(32):36466–74. 40852264 10.1021/acsomega.5c04995 PMC12368824

[195] Oliyaei N, Moosavi-Nasab M, Mazloomi SM. Therapeutic activity of fucoidan and carrageenan as marine algal polysaccharides against viruses. 3 Biotech. 2022;12(7):154. 35765662 10.1007/s13205-022-03210-6 PMC9233728

[196] Sigamani S, Venkatachalam SK, Digala P, Santhoshkumar M, Dharmaraj S, Duraisamy N, et al. Seaweed-derived polysaccharides: Multifunctional biomaterials for gut health and wound healing applications. J Functional Foods. 2025;134:107045.

[197] Howell M, Wang C, Mahmoud A, Hellermann G, Mohapatra SS, Mohapatra S. Dual-function theranostic nanoparticles for drug delivery and medical imaging contrast: perspectives and challenges for use in lung diseases. Drug Deliv Transl Res. 2013;3(4):352–63. 23936754 10.1007/s13346-013-0132-4 PMC3736595

[198] Setua S, Jaggi M, Yallapu MM, Chauhan SC, Danilushkina A, Lee H, et al. Chapter 6-Targeted and theranostic applications for nanotechnologies in medicine. In: Uskoković DP, editor., et al., Uskoković V. Nanotechnologies in preventive and regenerative medicine: Elsevier; 2018. p. 399–511.

[199] Martínez-Chávez LA, Hernández-Ramírez MY, Feregrino-Pérez AA, Esquivel EK. Cutting-edge strategies to enhance bioactive compound production in plants: potential value of integration of elicitation, metabolic engineering, and green nanotechnology. Agronomy. 2024;14(12):2822.

[200] Chan WY, Oakeshott JG, Buerger P, Edwards OR, van Oppen MJ. Adaptive responses of free-living and symbiotic microalgae to simulated future ocean conditions. Glob Change Biol. 2021;27(9):1737–54. 10.1111/gcb.15546 33547698

[201] Zhang S, Qamar SA, Junaid M, Munir B, Badar Q, Bilal M. Algal polysaccharides-based nanoparticles for targeted drug delivery applications. Starch-Stärke. 2022;74(7–8):2200014.

[202] McCall JR, Brown AP, Sausman KT, McCall IV SH. Microalgae nanotechnology and drug development. Handbook of Microbial Nanotechnology: Elsevier; 2022:169-90.

[203] Torres J, Costa I, Peixoto AF, Silva R, Sousa Lobo JM, Silva AC. Intranasal lipid nanoparticles containing bioactive compounds obtained from marine sources to manage neurodegenerative diseases. Pharmaceuticals. 2023;16(2):311. 37259454 10.3390/ph16020311 PMC9966140

[204] Mukherjee A, Sarkar D, Sasmal S. A review of green synthesis of metal nanoparticles using algae. Front Microbiol. 2021;12:693899. 34512571 10.3389/fmicb.2021.693899 PMC8427820

[205] Dutta V, Devasia J, Chauhan A, VL V, Jha A, Nizam A, et al. Photocatalytic nanomaterials: applications for remediation of toxic polycyclic aromatic hydrocarbons and green management. Chem Eng J Adv. 2022;11:100353.

[206] Bhardwaj R, Sahoo A, Yadav A, Nitesh, Sharma S, Meena M, et al. Economic perspectives of algal biotechnology. Industrial and biotechnological applications of algae: Springer; 2025:285–303.

[207] Khatoon UT, Velidandi A. An overview on the role of government initiatives in nanotechnology innovation for sustainable economic development and research progress. Sustainability. 2025;17(3):1250.

